# Omura’s whales (*Balaenoptera omurai*) off northwest Madagascar: ecology, behaviour and conservation needs

**DOI:** 10.1098/rsos.150301

**Published:** 2015-10-14

**Authors:** Salvatore Cerchio, Boris Andrianantenaina, Alec Lindsay, Melinda Rekdahl, Norbert Andrianarivelo, Tahina Rasoloarijao

**Affiliations:** 1 New England Aquarium, Boston, MA, USA; 2 Woods Hole Oceanographic Institution, Woods Hole, MA, USA; 3 Institut Halieutique et des Sciences Marines, Universite de Toliara, Toliara, Madagascar; 4 Biology Department, Northern Michigan University, Marquette, MI, USA; 5 Wildlife Conservation Society, Ocean Giants Program, Bronx, NY, USA

**Keywords:** Omura’s whale, *Balaenoptera omurai*, distribution, feeding ecology, breeding ecology, acoustic behaviour

## Abstract

The Omura’s whale (*Balaenoptera omurai*) was described as a new species in 2003 and then soon after as an ancient lineage basal to a Bryde’s/sei whale clade. Currently known only from whaling and stranding specimens primarily from the western Pacific and eastern Indian Oceans, there exist no confirmed field observations or ecological/behavioural data. Here we present, to our knowledge, the first genetically confirmed documentation of living Omura’s whales including descriptions of basic ecology and behaviour from northwestern Madagascar. Species identification was confirmed through molecular phylogenetic analyses of biopsies collected from 18 adult animals. All individuals shared a single haplotype in a 402 bp sequence of mtDNA control region, suggesting low diversity and a potentially small population. Sightings of 44 groups indicated preference for shallow-water shelf habitat with sea surface temperature between 27.4°C and 30.2°C. Frequent observations were made of lunge feeding, possibly on zooplankton. Observations of four mothers with young calves, and recordings of a song-like vocalization probably indicate reproductive behaviour. Social organization consisted of loose aggregations of predominantly unassociated single individuals spatially and temporally clustered. Photographic recapture of a female re-sighted the following year with a young calf suggests site fidelity or a resident population. Our results demonstrate that the species is a tropical whale without segregation of feeding and breeding habitat, and is probably non-migratory; our data extend the range of this poorly studied whale into the western Indian Ocean. Exclusive range restriction to tropical waters is rare among baleen whale species, except for the various forms of Bryde’s whales and Omura’s whales. Thus, the discovery of a tractable population of Omura’s whales in the tropics presents an opportunity for understanding the ecological factors driving potential convergence of life-history patterns with the distantly related Bryde’s whales.

## 1. Introduction

The taxonomy of Balaenopteridae, particularly involving what has been considered the ‘Bryde’s whale group’, is currently the focus of ongoing investigation and new discoveries [1–4]. The Omura’s whale (*Balaenoptera omurai*) was initially confused with and classified as Bryde’s whale (*Balaenoptera edeni*) during research whaling operations which took 459 specimens in the western Pacific and east Indian Oceans during the 1970s [5]; however, eight of these whales were later described as a distinct species and an ancient lineage basal to a *B. edeni*/*Balaenoptera borealis* clade [1,2]. In 2003, Wada *et al.* [1] described *B. omurai* as a distinct species, in part, based upon molecular phylogenetic evidence using mitochondrial DNA (mtDNA) complete control region sequences; their analysis grouped eight research whaling specimens (previously considered ‘pygmy Bryde’s whales’) taken near the Solomon Islands in 1976 and offshore of the Cocos (Keeling) Islands (in the east Indian Ocean) in 1978, with a 1998 stranding in the Sea of Japan as distinct from all other *Balaenoptera* spp. Sasaki *et al.* [2] later described in greater detail the molecular phylogenetic placement of the species using the complete mtDNA genome and short interspersed repetitive elements, establishing it as an ancient lineage with an estimated divergence time of 17.0±2.7 Ma from the *B. edeni*/*B. borealis* clade (although a more shallow divergence time of 9.13 or 9.75 Ma has recently been reported [6]). Wada *et al.* [1] also established a set of diagnostic skeletal features, particularly involving skull characters, which were later used to diagnose species identity using remains from whaling operations and strandings in several locales [7–9].

Prior to the definition of the species by Wada *et al.* [1], the lumping of existing specimens with *B. edeni* created some confusion and debate in the literature. Several studies investigating Bryde’s whales and the taxonomy of Balaenopteridae had noted marked discrepancies in both morphological characters and molecular genetic phylogenies for specimens of alleged Bryde’s whales from certain locations. Using allozymes, Wada & Numachi [10] first noted a phylogenetic placement of ‘pygmy Bryde’s whales’ from the Solomon Islands and Cocos Islands research whaling catches as basal to a clade formed by *B. borealis* and other Pacific and Indian Ocean *B. edeni*. A similar relationship was found by Yoshida & Kato [11] for the same Solomon Island samples using mtDNA partial control region and cytochrome *b*. Both studies suggested that these ‘pygmy Bryde’s whales’ were differentiated at the species level, and not simply a small-form or local stock of Bryde’s whales. Examination of 23 skulls taken in Philippine shore-based whaling operations in the Bohol Sea during the 1980s suggested that the specimens were atypically smaller than *B. edeni* skulls [12]; detailed morphological measures of 24 skulls from the same whaling operations later indicated they were in fact *B. omurai* [8]. Analysis of mtDNA cytochrome *b* sequences by LeDuc & Dizon [13] using a single genetic sample from the same Philippine whaling collection indicated phylogenetic position of the sample basal to *B. edeni*/*B. borealis*; this Philippine sample was separately deduced to be *B. omurai* based on the published phylogenies [2]. Since the establishment of Omura’s whales as a distinct species by Wada *et al.* [1] and Sasaki *et al.* [2], several molecular systematic studies focusing on relationships among Bryde’s whales (*B. edeni edeni* and *B. edeni brydei*, or alternatively *B. edeni* and *Balaenoptera brydei*) have independently affirmed the phylogenetic relationships described above using the same *B. omurai* sequences available on GenBank and expanded Bryde’s whales geographical sampling, with varied reconstruction methods and sets of markers [3,4]. We acknowledge that there is ongoing debate regarding the relationships and nomenclature among species labelled as Bryde’s or Eden’s whale, but consider that debate as independent from matters relating to Omura’s whale and do not here make any inference on the taxonomy of Bryde’s whales; throughout we will refer to Bryde’s whales as a group as *B. edeni*, with the exception of the case of GenBank samples that are labelled *B. brydei*, for which we maintain that nomenclature to avoid confusion. Towards resolving the relationships within this group, we believe that sequencing of bone material from the holotype specimen of *B. edeni* (Calcutta Museum) is fundamental to the understanding of these taxa and thus strongly recommended as advised by Reeves *et al.* [14].

Wada *et al.* [1] described the external appearance of *B. omurai* based upon very limited information from the Japanese research whaling expeditions in the Solomon and Cocos Islands, and the Sea of Japan holotype stranding specimen. It was stated that *B. omurai* has a similar appearance to the fin whale (*Balaenoptera physalus*) because of asymmetrical pigmentation with the right lower jaw and ventral grooves being white and the left lower jaw darkly pigmented, along with the pectoral fin being white dorsally, as well as ventrally on the anterior edge from shoulder to tip. Wada *et al.* [1] noted that the left gape of a Solomon’s Island animal was white, but there is no information regarding the right gape. The ventral grooves extend beyond the umbilicus and number approximately 80–90, which is diagnostic in comparison to Bryde’s whales having 42–54 grooves, at least for *B*. (*edeni*) *brydei* [15], but unknown for *B. edeni edeni*. The lateral rostral ridges that are prominent in Bryde’s whales are reported absent in *B. omurai*, however, Wada *et al.* [1] states that the head is not entirely smooth. The dorsal fin is reported to be small and falcate relative to Bryde’s whales and sei whales [16]. Reported sizes were smaller than most Bryde’s whales (hence the original common name ‘pygmy Bryde’s whale’) at less than 12 m, with reportedly physically mature individuals ranging from 10.3 to 11.5 m for females, and 9.6 to 10.0 m for males [1]; additionally, a smaller female at 10.1 m was reported to be sexually mature but not physically mature [1].

Virtually nothing is known about the ecology and life history of *B. omurai* because of a complete lack of confirmed live sightings. Wada *et al.* [1] reports that a 10.1 m female with 18 growth layers in its earplug (inferring 18 years of age, although not explicitly stated) was sexually mature and reproductive with three corpora albicantia in the ovaries, but not yet physically mature having an epiphysis of the sixth thoracic vertebra not fused with thin cartilage. A 9.6 m male with 38 growth layers had testes of 1.9 and 1.7 kg, and was physically mature with epiphyseal fusion showing a visible join [1]. Regarding timing of reproduction, none of five sexually mature females taken during research whaling in late October or mid-November were pregnant, although two had corpora lutea suggesting recent ovulation [1], and a neonate was recorded in August at 3 m length [16]. Regarding feeding habits, both crustacean and piscine remains were found in the stomach of a 7 m female from the Seto Inland Sea [16], and all specimens taken near the Solomon and Cocos Islands were reported to have fed only on *Euphausia diomedeae* [17,18].

Currently, the only confirmed specimens of *B. omurai* are the following from Japanese research whaling, Philippines shore-based whaling, and strandings: published genetic data from the Sea of Japan, Solomon Sea, Cocos Islands [1,2], Exmouth, northwest Australia [19] and Mauritania [20]; reported genetic data from Hong Kong (R. L. Brownell Jr and R. Brown, 19 May 2015, personal communication; http://hkmarinelife.hk/2014/12/23/taipo-whale-identified-as-recently-discovered-omuras-whale/); and skull morphology from Taiwan [7], Thailand [7], the Philippines [8], Malaysia [9] and Gulf St Vincent, South Australia [21]. There are reported sightings of live animals with photographic evidence from Thailand [16,22], New Caledonia [23,24] and the Lacepede Islands, northwest Australia (http://www.pbase.com/wildlifeimages/omuras_whale). However, there are to date no field observations confirmed through genetic analysis of biopsies for live animals. Thus, there is a complete absence of ecological and behavioural data, or even detailed description of living animals.

Here we present, to our knowledge, the first detailed documentation of Omura’s whale in the field, reporting on a population off the coast of northwest Madagascar, with initial observations of the ecology, behaviour and bioacoustics of the species. The discovery of this population also represents the first documentation of the species in the western Indian Ocean.

## 2. Methods

### 2.1 Study site and boat surveys

**Figure 1.**
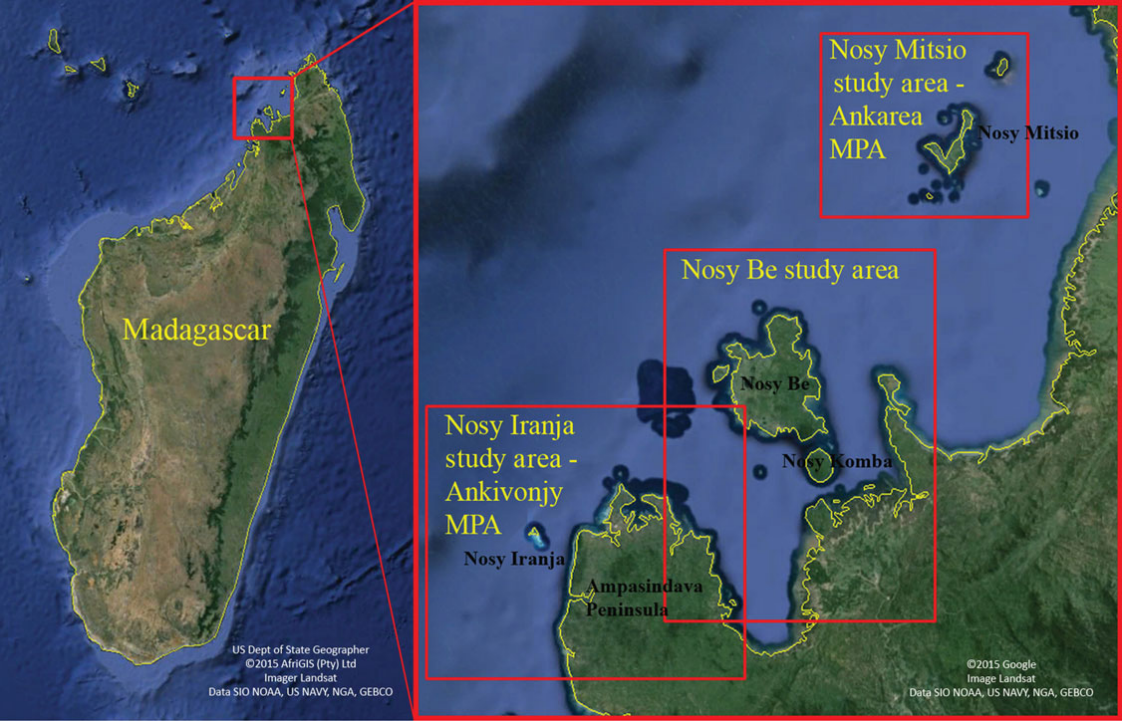
Location of the study site off northwest coast of Madagascar, including the Nosy Be and Nosy Iranja study areas.

Between 2007 and 2014, cetacean populations were assessed on the northwest coast of Madagascar including the Nosy Be and Nosy Iranja/Ampasindava peninsula regions (figure 1). The objective was to gain a better understanding of the diversity, distribution and relative abundance of cetaceans, initially with a focus on coastal dolphins [25]. Effort from 2007 to 2011 focused exclusively on the near coastal waters of the Nosy Be region. Effort from 2012 to 2014 was shifted to the Ankivonjy Marine Protected Area (MPA) covering 196 659 ha approximately 50 km southwest of Nosy Be, and encompassing the Nosy Iranja/Ampasindava Peninsula regions (figure 1). Surveys in this area covered more diverse habitat, including coastal, shallow open shelf, shelf break slope and deep offshore waters.

Boat surveys were conducted from an 8–10 m outboard boat with two to four observers for approximately one month each year, between July and December. To survey along the coastal zone (particularly around Nosy Be, 2007–2012), transects parallel to the coasts were employed to effectively cover shallow-water coastal habitat. For deeper water surveys off Nosy Iranja, saw-tooth or box pattern transects were conducted crossing the bathymetric gradient out to 2000 m in order to maximize habitat coverage. Complete tracks of daily boat movements were logged on handheld GPS receivers, and precise records of effort were made throughout the day, so as to calculate standardized sighting per unit effort (SPUE) measures. Working time on the boat was stratified by distinct effort categories including three types of active search efforts: (i) in very shallow coastal waters (in close proximity to the coast line and generally shallower than 20 m), (ii) in shallow open shelf waters (generally greater than 10 m and less than 100 m), and (iii) in deep offshore waters (past the shelf break, generally greater than 100 m up to 2000 m; exclusively off Nosy Iranja). When groups of cetaceans were encountered, the species was identified, and individual identification photographs obtained of each identifying feature (right and left side pigmentation pattern and dorsal fin for Omura’s whales) from each individual using digital SLR cameras fitted with zoom lenses (70–200 mm). Underwater video was obtained using a Contour ROAM2 HD Camcorder (1080p resolution), held over the side of the boat during close encounters with whales. Once photographs were obtained, skin biopsies of individuals were collected whenever possible, using either a lightweight crossbow (150 lb prod) or a compressed CO_2_ rifle equipped with an adjustable pressure valve, and a biopsy dart designed to penetrate the epidermis and retain a 0.8×2 cm plug of skin which was preserved in 90% EtOH. To infer depth of sightings, bathymetric data were downloaded from the 2014 GEBCO dataset (www.gebco.net) in the form of a 1 arc second raster format. Because GEBCO data were lacking the granularity needed for depth analysis in near shore waters, sightings depths were derived from digital scans of marine navigational charts. The charts were georeferenced before their features were digitized, and a bathymetric raster was then interpolated from those features using ArcGIS 10.2 Topo to Raster tool. To estimate sea surface temperature (SST) at encounter positions, daily blended global 1 km sea surface temperature (G1SST) images [26] were downloaded from the NASA Jet Propulsion Laboratory (JPL) Regional Ocean Modeling System group (http://podaac-ftp.jpl.nasa.gov/OceanTemperature/ghrsst/data/L4/GLOB/JPL_OUROCEAN/G1SST/).

### 2.2 Photographic data

Photographic identification data were processed using custom protocols developed for comparison of identification (ID)-photos of humpback whales [27]. The protocols were modified to accommodate Omura’s whale individual identification using the blaze/chevron pigmentation patterns and dorsal fins. A photographic database was developed for the 2011–2014 Omura’s whale data, and initial comparisons of photos indicate that variation in pigmentation patterns and dorsal fin shape and markings is sufficient for confident individual identification for good quality photographs.

### 2.3 DNA sequence acquisition

DNA was extracted from tissue samples (less than 25 mg) using a DNEasy Tissue Extraction Kit (Qiagen, Valencia, CA, USA) according to the manufacturer’s protocol, and extracts were quantified using a Nanodrop 2000 spectrophotometer. Total genomic DNA concentrations averaged 151.26 (±63.24) ng μl^−1^. Mitochondrial control region fragments were amplified with primers Dlp-10/Dlp-5 [28], using Phusion Kits (Fisher Scientific), with 10 ng of template and primer concentrations of 5 μM. Thermal profiles were designed as recommended by the manufacturer (Fisher Scientific): 30 cycles of 98°C denaturation (20 s), 59°C annealing (20 s) and 72°C extension (30 s). PCR products were isolated, visualized and extracted (Qiagen PCR Gel Extraction Kit) from 1% low-melt agarose gels run with 1× Lithium-Borate EDTA buffer. Forward and reverse strands of PCR products were directly sequenced on an ABI 3730xl DNA Analyzer (GeneWiz Inc. http://www.genewiz.com/), primers were trimmed from all sequences, and sequences were reconciled and analysed using Geneious v. 7.1.5 [29]. Reconciliation included visual inspection of forward and reverse strands sequenced from the same fragments, where discrepancies were called in favour of the higher quality base. Between species number of sequence differences and net nucleotide substitutions per site (*D*_*A*_ [30]) were calculated in DNAsp [31].

### 2.4 Phylogenetic analysis

For phylogenetic analyses, we included sequences available on GenBank for five congeneric *Balaenoptera* species, including *B. omurai*, and used *Eubalaena australis* as an outgroup (see the electronic supplementary material, table S1 for sample information). Five existing *B. omurai* sequences downloaded from GenBank were used in the analysis: two from Japan (Tsunoshima Island, Sea of Japan [1]; Shikoku, Kagawa Prefecture, Inland Sea [2]), one from the Solomon Islands (10°03′ S, 157°29′ E [1]), one from the Cocos Islands (10°51′ S, 97°02′ E [1]), and one from China (Rui’an City, East China Sea [32]). Sequences were aligned using ClustalW (IUB cost code, gap open cost =15, gap extend cost =6.66) as executed in Geneious v. 7.1.5. Evolutionary relationships were assessed using three different methods: maximum-parsimony, maximum-likelihood and Bayesian inference. Trees were reconstructed using maximum-parsimony in PAUP* [33], using a heuristic search of treespace, outgroup rooting with stepwise addition of taxa and no other assumptions. Branch support was evaluated with 1000 bootstrap replicates. PHYML [34] was used for maximum-likelihood estimation of relationships, assuming a HKY+*Γ* model of sequence evolution (best model identified by Jmodeltest2 [35,36]), with model parameters estimated from the data. Branch support was evaluated using 1000 bootstrap replicates. Bayesian analyses were performed using MrBayes [37], assuming a HKY+*Γ* model of sequence evolution with unconstrained branch lengths. Tree/parameter space was sampled every 200 iterations when searched in a Markov chain Monte Carlo chain length of 1.1 M (100 k burn-in) with four heated chains (*temp*=0.2). Convergence of posterior split probabilities and branch lengths was examined and confirmed graphically using the AWTY program [38].

### 2.5 Acoustic data

Recordings were collected with a single hydrophone suspended from the boat using a Cetacean Research^TM^ CR-1 hydrophone (±3 dB from 16 Hz to 48 kHz, sensitivity of −198 dB re: 1 V μPa^−1^) with variable gain pre-amplifier connected to a solid-state recorder (24 bit, 96 kHz sample rate). Recordings were made either when there was positive visual confirmation of an Omura’s whale in close proximity to the boat, or at predefined recording stations throughout the study region for 10–15 min during boat surveys. In addition, three SoundTrap 202 archival recorders (www.OceanInstruments.co.nz, ±3 dB from 20 Hz to 60 kHz, noise floor 35 dB re uPa/root-Hz) were deployed three times between 26 October and 15 November 2014, for 4–9 days per deployment (16 bit, 96 kHz sample rate) at one of four different sites spanning 55 km of the study area. Data were down-sampled from 96 kHz to 4 kHz and manually browsed for the presence of low frequency baleen whale calls potentially produced by Omura’s whales using Raven Pro 1.4 (www.birds.cornell.edu/brp/raven/RavenOverview). Spectrograms (2048pt FFT, 75% overlap) were browsed in 150 s windows in the 0–350 Hz bandwidth for all boat-based recordings, and a portion of the SoundTrap recordings from each recording location.

## 3. Results

During surveys in the Nosy Be region, we first sighted a small rorqual species on 5 August 2011, involving an encounter with a mother–calf pair, and then later in the day a single adult. The following year, a loose aggregation of four individuals (determined through photographic identification) was encountered on 8 December 2012. In each case, we initially thought them to be Bryde’s whales. The asymmetrical coloration of the head was not noticed or photographically documented at this time, and the presence of light pigmentation was noted, but considered a possible variation in Bryde’s whales as they are poorly known from Madagascar [39]. A shift in focal study range and habitat to the shelf waters inshore of Nosy Iranja led to extensive observations of the small rorqual during 2013 and 2014. Photographic evidence indicated that their external appearance matched that reported for *B. omurai* (detailed below). Species identity was confirmed with genetic data using DNA extracted from 23 biopsy samples from 20 individuals (18 adults and two calves whose mothers were also sampled) with the same external appearance.

### 3.1 Genetic sequence analysis and species identification

**Figure 2.**
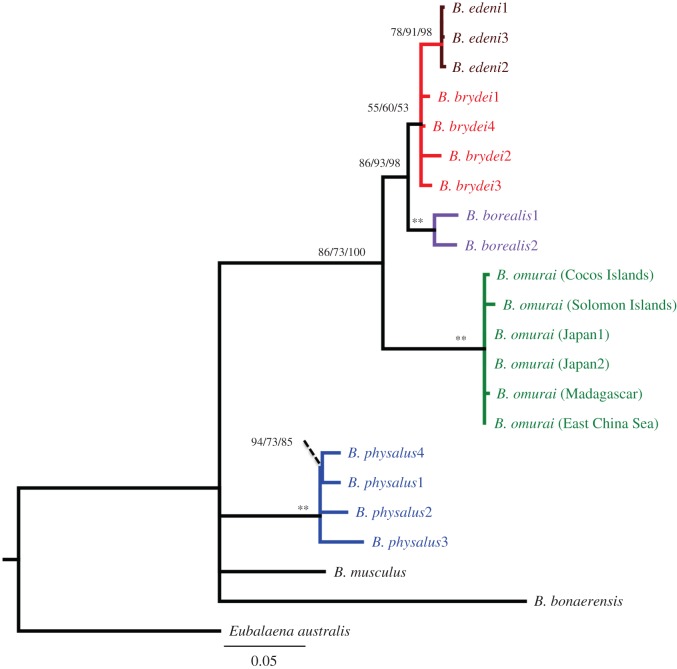
Phylogeny of *Balaenoptera* species, indicating placement of Madagascar *B. omurai* samples among known available *B. omurai* sequences from the western Pacific and eastern Indian Oceans. Topology is a maximum-likelihood tree inferred from partial mitochondrial control region sequences. Symbols near nodes refer to bootstrap support (BS) scores and posterior probabilities (PP), for maximum-likelihood, maximum-parsimony and Bayesian analyses, respectively (BS/BS/PP). A double asterisk indicates nodes with greater than 90% BS and greater than 0.95 PP. Other scores are represented as BS/BS/PP. Scale bar shows estimated changes per site.

We reconciled double-stranded mitochondrial partial control region sequences from 19 of the 23 Madagascar samples. The remaining four samples returned high-quality scores (*QS*>50) for one of the two strands (forward or reverse), which was used in the analysis. The aligned 398 base pairs (bp) for all 23 samples were identical sequences representing a single identified haplotype for the partial control region sequence (GenBank no. KT582064; alignment available at http://commons.nmu.edu/facwork_datasets/1/), so only one was included in the phylogenetic analysis. The Madagascar haplotype matched previously published mitochondrial control region sequences from *B. omurai* with 99.7–99.9% identity, differing from existing *B. omurai* sequences by one base (Japan [1,2] and East China Sea [32]), two bases (Cocos Islands [1]) or three bases (Solomon Islands [1]) within the aligned 398 bp sequence (table 1). The final alignment used for phylogenetic reconstruction of 22 Mysticete sequences included 402 aligned characters. Optimal trees from all three analyses had the same basic branching topology, and the Madagascar haplotype was embedded in a monophyletic group with all previously published *B. omurai* samples (figure 2). The *B. omurai* clade formed a sister clade to a *B. edeni*/*B. borealis* clade, as previously documented [1,2]. Therefore, we are confident that this represents the first documentation of *B. omurai* in the West Indian Ocean, and most extensive field documentation to date.

**Table 1.** Pairwise sequence differences between and within species. (Between species number of sequence differences and net nucleotide substitutions per site (*D*_*A*_) are shown above and below the diagonal, respectively. Number of samples of each species shown in the left column.)

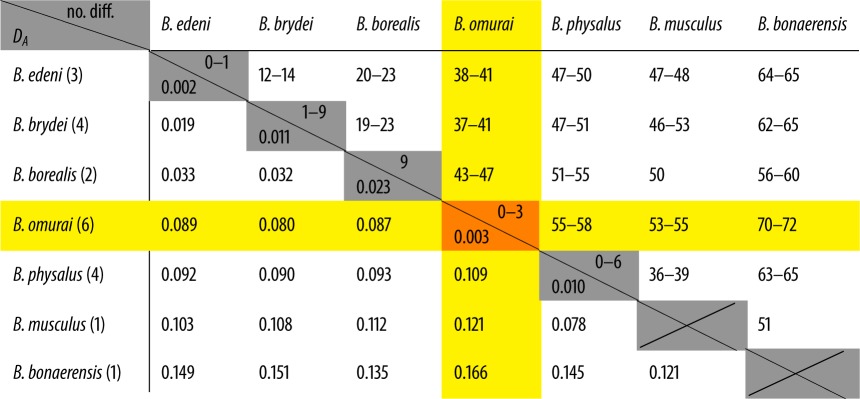

### 3.2 External appearance

**Figure 3.**
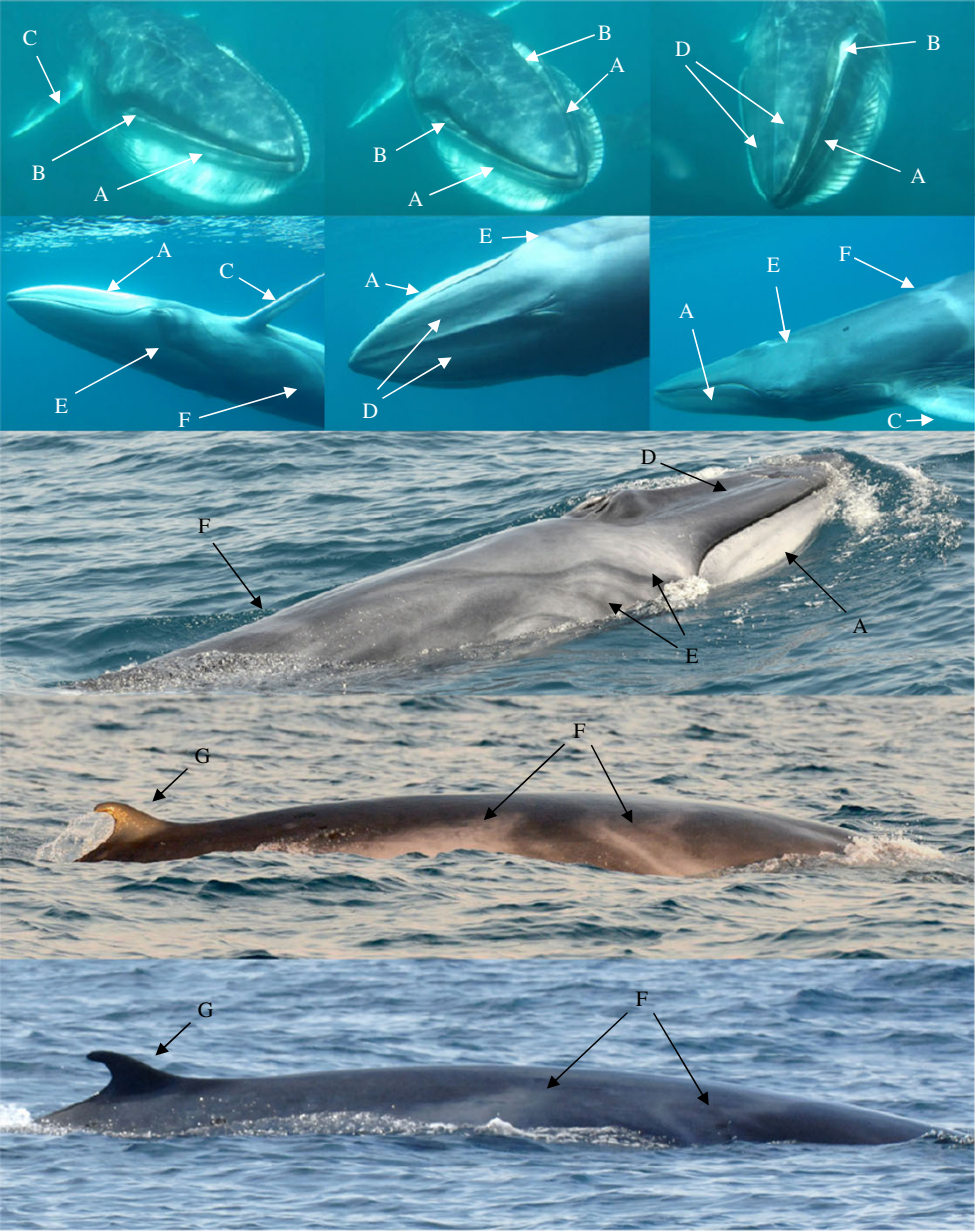
Images of Madagascar *Balaenoptera omurai* displaying details of pigmentation and external appearance. Five different individuals are pictured: underwater video frame captures of an adult female with calf sighted on 9 November 2013 that had just completed a feeding lunge (first row), and a lone adult on 22 October 2014 (second row), and above surface photographs from three adults sighted on 13 November 2014 (third row), 9 November 2013 (fourth row) and 12 December 2012 (fifth row). Visible features: (A) asymmetrical coloration of the lower jaw, with lightly pigmented right jaw and darkly pigmented left jaw; (B) asymmetrical coloration of the gape (inferred by inner lower lip), with lightly pigmented left gape and darkly pigmented right gape; (C) leading edge of pectoral fin white from tip to shoulder; (D) the apparent absence of lateral rostral ridges, with only faint indications detectable at some angles; (E) lightly pigmented blaze originating anterior to the eye, present only on the right side, with dark eye and ear stripe, two additional dark stripes and a light inter-stripe wash; (F) lightly pigmented chevron anterior to dorsal fin, present on both sides but asymmetrical and most prominent on right where it displays a double banded pattern; (G) highly falcate dorsal fin with gradual sloping insertion into dorsum.

**Figure 4.**
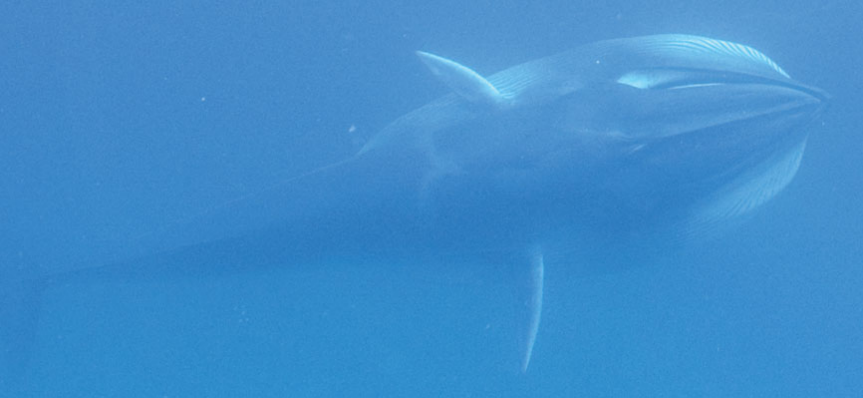
An Omura’s whale subsurface lunge feeding, displaying several of the features detailed in figure 3.

Extensive photographic data from 44 sightings combined with high-quality underwater video of three individuals provided a detailed description of the species external appearance, congruent with what has been reported previously from animals in the eastern portion of the species range [1,16]. Size approximation of individuals ranged from 8 to 12 m based on comparison with our either 8 m or 9 m boat during very close approaches (within 2–3 m of boat). Calves were estimated at 3–5 m in length or about half the size of the boat. In general, the body pigmentation is extremely similar to that of *B. physalus* [40]. All individuals, including calves were strongly asymmetrically pigmented, with the right lower jaw being white to very pale, and the left lower jaw being dark (figure 3, feature A). Underwater photographs and video stills of two lunge feeding individuals indicated that the gape, as inferred by the inner lower lip, is also asymmetrically pigmented but in reverse of the jaw, with the left side being white and right side dark (figure 3, feature B; figure 4). The anterior edges of the pectoral fins are white (figure 3, feature C), with the white being more extensive on the right pectoral fin where it gradually grades into darker pigmentation.

White pigmentation is generally more extensive on the right side of the body and also present on the left. The right side displays a lightly pigmented blaze that starts from the jaw anterior to the eye, sweeping over the eye and across the back; posterior to this blaze, there is a dark eye strip, and a light inter-stripe wash that is bisected by three additional dark stripes, all sweeping back over the right pectoral fin (figure 3, feature E; electronic supplementary material, figure S1). These blaze and stripe patterns are not evident on the left side; however, some individuals have a light wash that extends back from posterior to the nares on both sides. Posterior to the pectoral fin there is a light pigmented chevron with a double banded pattern that sweeps forward from mid-back in a V-shape that is strongly asymmetric when viewed dorsally (figure 3, feature F; figure 4; electronic supplementary material, figure S2). Clarity of the pigmentation pattern in photographs varied with lighting and photographic quality, but good photographs and clear field observations indicated that all individuals consistently displayed the described pigmentation features, despite individual variability in the pattern (electronic supplementary material, figures S1 and S2).

The dorsal surface of the rostrum has a single strong medial ridge, and the absence of pronounced lateral ridges that are diagnostic to Bryde’s whales; however, there are faint lateral ridges and other ripples in the dorsal surface of the rostrum that are visible only with some viewing angles and lighting (figure 3, feature D). Good photographs of young calves indicated that these lateral ridges were more pronounced and obvious in these immature individuals, suggesting that there is variation in the rostrum appearance during development. On one occasion, an adult rolled within 2–3 m of the boat (the event also captured on underwater video in figure 3, second row), allowing good photographs of the anterior ventral surface as it broke the water; 32 ventral grooves were exposed extending from the pectoral fin to approximately one-third to maximum one-half of the belly circumference, suggesting throat grooves numbering between 65 and 95. The dorsal fin tends to be strongly falcate and displays an anterior insertion into the dorsum with a gradual slope as opposed to a sharp right angle (figure 3, feature G), mid-way in character between *B. physalus* (which has a more gradual sloping insertion) and *B. edeni* or *B. borealis* (which have a sharper angle of insertion). The dorsal fin also tends to be proportionally smaller and less upright than these other species. Individuals’ dorsal fin shape can vary from moderately recurved to nearly triangular (electronic supplementary material, figure S3). During surfacing the dorsal fin typically emerges after the head and blowhole shield submerge, so that the two are not seen at the surface together, and the flukes were never observed to be lifted above the surface upon diving. There was no evidence of cookie cutter shark (*Isistius* sp.) scars, unlike populations of Bryde’s and sei whales that range in the deep offshore waters off the west coast of southern Africa [39]. There were numerous faint rectangular marks, both darker and lighter than the skin pigmentation, with a ‘cloven hoof’ appearance, that may represent attachment sites of remora (Echeneidae). However, no remoras were ever observed attached to individual *B. omurai* (although they are present in the area seen with other cetaceans and whale sharks, *Rhincodon typus*). It is not clear whether these marks represent permanent scarring, but it seems unlikely.

### 3.3 Sighting characteristics, habitat and behaviour

**Figure 5.**
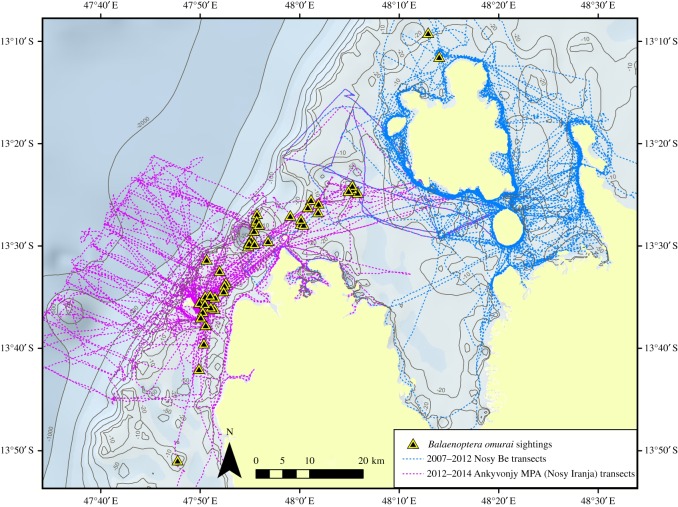
Distribution of sightings of *Balaenoptera omurai* within the northwest Madagascar study site. Tracks during the two different phases of the project, based off Nosy Be and Nosy Iranja, respectively, are shown by colour.

Between 2011 and 2014, there were 44 sightings of *B. omurai* groups, primarily during 2013 (13 groups) and 2014 (25 groups) when based from Nosy Iranja and working the shallow open shelf waters off the Ampasindava Peninsula (figure 5). During coastal surveys focusing on dolphins in the Nosy Be area from 2007 to 2012 (figure 5, blue tracks), 250.5 h search effort were conducted in coastal habitat with no sightings of *B. omurai*, and 102.7 h in open shelf habitat with only two sightings on the extreme north and outer shelf of Nosy Be (figure 5, blue tracks). During surveys off Nosy Iranja from 2012 to 2014 (figure 5, magenta tracks) with effort in all three habitat types, there were 47.3 h of search effort in coastal habitat, 111.8 h in open shelf habitat and 99.5 h in deep offshore habitat; all sightings (42) were made on the open shelf inside Nosy Iranja and outside or north of the Ampasindava Peninsula (figure 5, magenta track). SPUE calculations in the Nosy Iranja study range are, therefore, 0.38 grps hr^−1^ search for the shallow open shelf, as opposed to less than 0.02 grps hr^−1^ for coastal waters and less than 0.01 grps hr^−1^ for deep offshore waters. This suggests that at least in this region and time of year, *B. omurai* occurs strictly in the shallow shelf region inside the shelf break, avoiding both deep water off the shelf and very shallow coastal waters and embayments. Depth of encountered groups ranged from 4 m to 202 m , with an inter-quartile range of 10–24 m , a median depth of 13.5 m and mean of 31.0 m (s.d. 48 m). During our encounters the SST ranged from 27.4 to 30.2°C with a mean of 28.6°C (s.d. 0.68°C), as estimated from the JPL global daily blended remote sensing data for the 1 km block in which the sighting occurred.

**Figure 6.**
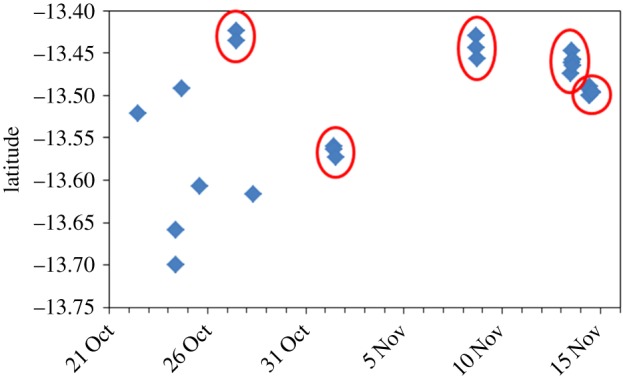
Spatial and temporal distribution of *Balaenoptera omurai* sightings in 2014, showing clustered tendency of loose aggregations of individuals interspersed with periods without sightings despite consistent survey effort throughout the field season.

There were never more than two individuals in a group, when strictly defined as a number of individuals within several body lengths of each other. Of eight groups with two individuals, six were mother–calf pairs (of four different mothers) and only two were pairs of adult-sized individuals. In both cases of pairs, the association was brief, on the order of 10 min or less. The remaining 36 encounters were with single individuals. Mean group size was thus small at 1.2 (s.d. 0.4) individuals. Despite this apparent tendency for singletons, whales were often encountered in loose aggregations, so that when one individual was encountered there were often several others (up to six documented) within a few to several hundred metres of each other. Thus, it is possible that these apparently unassociated individuals (based on a strict definition of a ‘group’) may in fact be interacting acoustically, raising questions in regards to how to define a group for this species. On one occasion on 14 November 2014, after encountering one individual, we spent 4 h within an approximately 1×2 km area, working with a minimum of six different individuals that appeared to maintain their relative positions. Whereas feeding was frequently observed at other times, overt surface lunge feeding was not observed during encounters with individuals in this aggregation. All individuals appeared to be approximately 100–300 m apart from the next closest, and it was during these encounters that one of the briefly associating pairs was observed, in addition to breaching behaviour. Also, sightings across the field seasons were not consistent over time, so that whales would be encountered for a few days, and then seemingly absent from the study area for several days despite consistent survey effort. Thus, it appeared that *B. omurai* distribution was spatially and temporally clustered (figure 6), as loose aggregations of individuals moved through the region, apparently ranging beyond the study area. A whale-watch partner in our study area reported seeing aggregations of these ‘petite rorquals’ throughout the year, while operating small boats from April to December (T. Guillemain d’Echon 15 November 2014, personal communication).

Sightings included four different mothers with calves, photographically identified by dorsal fin and pigmentation pattern, one in August 2011, and three in November 2013 (two of which were sighted on two occasions). Of the three calves sighted in 2013 (on 9, 10, 12 and 26 November), all had moderately to strongly bent dorsal fins, indicating that they were relatively young if not neonate, although fetal folds were never observed. This suggests that this region may serve as calving habitat, and that calves are born at least in the general period prior to November. The calf observed in August 2011 had an erect dorsal fin, however, it was still clearly a calf of the year and we believe it unlikely that is was born prior to November of the previous year, suggesting a protracted calving period. Subsequent to the data collection for this manuscript, one of us (B.A.) reported sighting two mother–calf pairs on 29 August 2015, in which the calves were substantially smaller than in previous years’ sightings (no more than 3 m) and probably neonates. There were no mother–calf pairs sighted in 2014, despite the most extensive search effort in shelf waters and the majority of group encounters (25 of the 43 groups, compared with 13 in 2013), suggesting that either reproductive output or timing varies among years. There was at least one recapture between years, notably of an adult female, sighted in 2012 in a loose aggregation of four whales, and then re-sighted in 2013 with a young calf. Therefore, at least some females appear either to be resident or have site fidelity to the area, and may probably be engaging in both mating and calving behaviours in this habitat.

There were also numerous, unambiguous observations of lunge feeding and defecation, encompassing greater than or equal to 50% of encounters. Observed surface lunge feeding involved a roll at the surface, with at least some individuals oriented with the right (lightly pigmented) side down, and the left pectoral fin and fluke extending above the surface. On no occasions during feeding events were small fishes observed in the water; during one encounter with a feeding whale, particularly clear observation of submerged lunge feeding was made and photographed (figure 4), and there were clearly no fishes seen. The upper water column appeared to have a relatively high opacity because of a yellowish particulate or small zooplankton; sampling equipment was not available to identify the source. During the same period in 2014, there was a relatively high density of whale sharks in the region, and local whale watchers reported association of sightings of *B. omurai* with sightings of whale sharks and schools of ‘Bonito’ (T. Guillemain d’Echon 15 November 2014, personal communication). Thus, it seems likely that these species are feeding on similar resources and that the *B. omurai* in this region and time may be feeding on an abundant zooplankter.

### 3.4 Acoustic data and vocal behaviour

**Figure 7.**
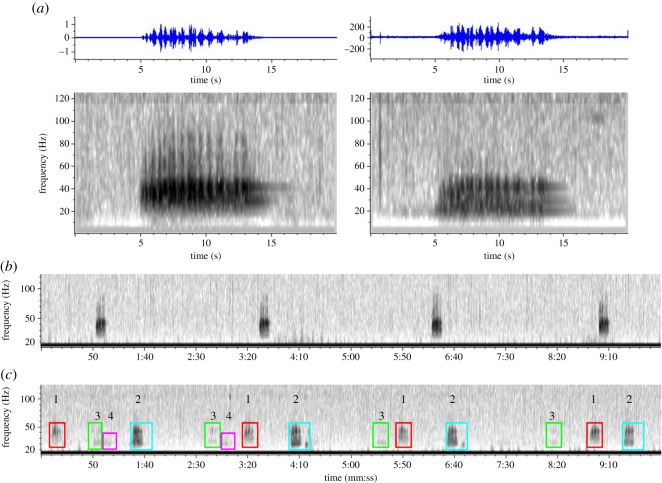
Spectrograms of recordings with calls attributed to *Balaenoptera omurai* showing (*a*) a stereotyped, amplitude-modulated pulsative call at 15–50 Hz, recorded on two separate days assumed to be from different individuals, (*b*) 10 min clip of a rhythmic series of calls with consistent 170–180 s repetition rate, interpreted as uttered by a single individual, and (*c*) 10 min clip of what appears to be a chorus of four individuals inferred from overlapping rhythmic series with different apparent received levels, including two animals in the foreground (labelled ‘1’, red and ‘2’, blue) and two animals in the background (labelled ‘3’, green and ‘4’, magenta); stereotyped, rhythmically repetitive vocalizations uttered in choruses of different individuals suggest song-like characteristics and function.

Boat-based recordings from 2013 and 2014 were examined manually, amounting to 14 h 57 min from 67 recordings on 13 days in 2013 and 10 days in 2014. On nine occasions, recordings were made in the presence of *B. omurai*, with recording generally starting within 50 m of the whale’s last known position. On five of those occasions, an amplitude-modulated (AM) low frequency vocalization was recorded (figure 7), on three occasions in 2013 and two occasions in 2014. The call was only recorded when *B. omurai* were sighted. Furthermore, no other baleen whales were in the vicinity of the recording station when the vocalization was recorded. There were a total of 44 calls attributed to Omura’s whales detected in the five recordings. The calls were long, broadband, AM in the 15–50 Hz bandwidth (figure 7*a*), and in each case uttered with a consistent rhythmic repetition every 2–3 min (figure 7*b*). For purposes of time/frequency measurements, 21 calls with a signal-to-noise ratio greater than 10 dB were used, measured with the Raven Pro 1.4 Average Power measurement in a 16–52 Hz bandwidth. Calls had an average duration of 9.2 s (s.d. 0.92 s), average peak frequency of 36.1 Hz (s.d. 6.19 Hz), average low frequency of 14.9 Hz (s.d. 5.40 Hz) and high frequency of 52.9 Hz (s.d. 2.19 Hz) and a 90% bandwidth of 25.8 Hz (s.d. 5.30 Hz). The repetition rate, measured as the inter-call interval, was 152.9 s (s.d. 16.47 s, *n*=37) for all sequential calls, and ranged from 134.4 s (s.d. 8.88 s, *n*=6) to 176.8 s (s.d. 32.01 s, *n*=3) within each recorded series. The pattern of pulses within individual calls appeared consistently recognizable and stereotyped across calls both within and between series, with the most obvious feature being a ‘doublet’ of strong pulses at the end of the call, following a brief low amplitude section of the call (figure 7*a*).

Preliminary analysis of long-term recordings from the SoundTrap recorders revealed these calls to be present at all of the four locations spread across 50 km from the most southern part of the study area to the area of concentration of sightings just north of the Ampasindava Peninsula (figure 5). In every instance, the calls were stereotyped in structure and uttered in rhythmically repetitive sequences (figure 7*b*), with series documented to go on uninterrupted for multiple hours. On at least 3 days of approximately six partial days that were examined, there were clearly multiple overlapping series, seemingly at different and consistent amplitudes (probably resulting from different distances and propagation characteristics of the sources), which we interpret as multiple individuals acoustically displaying in a chorus (figure 7*c*). It is noteworthy that there were no other baleen whale species sighted in the region at the densities suggested by this acoustic data, thus providing another indication to attribute these vocalizations and behaviour to *B. omurai*.

## 4. Discussion

Prior to our discovery of Omura’s whales in Madagascan waters, there was a complete absence of field data on this species. Here we have presented evidence supporting the species identification of the small rorqual that we have observed as *B. omurai*, along with details on the external body appearance, and initial observations on habitat use, local distribution, feeding ecology, reproductive ecology and vocal behaviour. Notably, this represents to our knowledge, the first identified population tractable for field study. In the absence of a detailed description of the external appearance of *B. omurai*, it has been difficult for field biologists to confidently distinguish the species from congeners, particularly Bryde’s whales. We believe that this difficulty was primarily because of the lack of observations, and now with the detailed description we have provided it should be no more difficult to distinguish Omura’s whales in the field than it would be to distinguish fin whales from sei whales, or sei whales from Bryde’s whales, provided adequate observations and documentation.

### 4.1 Species confirmation and genetic diversity

We consider this population of small rorquals off northwest Madagascar to be unequivocally *B. omurai* given: (i) the genetic distinctiveness of *B. omurai* relative to other balaenopterids [1,2]; (ii) the close similarity of the Madagascar sequences to *B. omurai* sequences from Japan, East China Sea, Solomon Islands and Cocos Islands; and (iii) the phylogenetic reconstruction that places the Madagascar sequences within the well-supported clade of other *B. omurai* sequences. The finding of a single mitochondrial control region haplotype among the 18 Madagascar adult individuals is noteworthy, along with the similarity to existing *B. omurai* sequences (differing by one to three bases). Similar low diversity has been reported in populations of coastal small-form Bryde’s whales, *B. edeni edeni*, in the Indian Ocean [3] from three separate study sites for a 299 bp control region fragment: among 29 samples from Bangladesh, and 16 samples from Oman, only a single haplotype was identified and shared by both sites (increasing to two haplotypes for a 407 bp fragment); the same haplotype was also found among 16 samples from Japan, along with two additional haplotypes. Rosel & Wilcox [4] also found extremely low diversity within a population of *B. edeni* in the Gulf of Mexico (GOMx), with only two haplotypes found in a 375 bp fragment of the mitochondrial control region and three haplotypes in the complete 936 bp control region among 23 samples, and similar low diversity for two other mitochondrial genes (*cytb* and *cox1*, each with no variability) and among 42 nuclear microsatellite loci. Rosel & Wilcox [4] also advocate that the GOMx population warrants a unique taxonomic status, with phylogenetic distinctiveness being on par with that observed between other *B. edeni edeni*, *B. edeni brydei* and *B. borealis*. Thus, among these studies, at least three phylogenetically distinct taxa (*B. edeni edeni*, *B. edeni* GOMx and *B. omurai*) have been identified with low population genetic diversity. Low diversity can indicate small effective population size and/or small, relatively recent founding events. Each of these three taxa potentially share key ecological characteristics of being tropical/subtropical baleen whales, with likely non-migratory behaviour and small locally resident populations. We suggest that the observed low genetic diversity may be linked to these ecological and life-history similarities, and that further study should be conducted to explore the ecological parameters that may influence the convergence that has been documented in these geographically and phylogenetically disparate populations.

### 4.2 Distribution, habitat use and feeding ecology

Our sightings data indicate that in northwest Madagascar during the time of year in which we surveyed (October–November), *B. omurai* appears to be distributed exclusively on the shallow continental shelf habitat, within approximately 10–15 km of the shelf break and primarily 10–25 m depth. Yamada [16] suggested that the species is distributed in both shallow and deep water. The deep water observations were only off the Cocos Islands, and may be the case in the eastern part of the Indian Ocean from which the research whaling data come, as well as during other times of year or other parts of the southwest Indian Ocean. However, given our survey effort (figure 5), we are confident that the species did not occur off the shelf in deep water that we surveyed, or around Nosy Be and associated islands and coast within the extensive Nosy Be embayment. Feeding in this shelf habitat was frequently observed, and it seems likely that distribution is related to a patchy food resource, possibly zooplankton based upon our preliminary observations of never seeing fishes during feeding events. Lunge feeding on euphausids has been well documented in balaenopterids [41,42], but to our knowledge it is unclear if it has been documented on smaller zooplankton. Sei whales are known to both skim and lunge feed [43,44], and have been documented making slow lunges with a semi-distended throat on small unidentified zooplankton [45]. Prey sampling will be needed to determine what prey *B. omurai* are feeding on off Madagascar. Temporal distribution of sightings during the study period was also patchy, with long periods of days without seeing animals, while readily finding them during other periods. This suggests that the population is moving around within a range greater than our study area, and potentially shifting up and down the coast in the shelf habitat with food resources. Scuba dive tour operators have reported seeing what they interpreted as Bryde’s whales in aggregations around the Nosy Mitsio island group, approximately 50 km northeast of Nosy Be (figure 1). Although our data cover a relatively short period of the year, there is indication that the species may be present most of the year, with reports of similar-sized aggregations of individuals from April through to December. Year-round monitoring (e.g. through passive acoustics coupled with targeted field surveys) will be needed to discern temporal and seasonal distribution, and geographical extension of sampling effort north and south along the coast for defining spatial patterns. Studies on feeding ecology using field observations and stable isotopes are feasible given the accessibility of the population, and will inform the ecological parameters that influence spatial and temporal distribution.

It is unclear whether previously reported Bryde’s whales in the Madagascar region (or minke whales [46] for that matter) may represent misidentified sightings of *B. omurai*. Never during our surveys did we encounter a whale matching the typical morphology of a Bryde’s whale, and clearly all of our genetic samples were *B. omurai*. Best [39] reports on the most substantive study of Bryde’s whales in the southern African region, including a description of scientific catch data from the south of Madagascar (some 1500–2000 km from our study site) where 105 Bryde’s whales were taken in March 1977 [5,17]. Size of these animals was smaller than other southern African Bryde’s whales, even in comparison to the inshore small-form found off South Africa; however, at modes of 12.2 m for males and 13.1 m for females, these whales were somewhat larger than reported for *B. omurai* in the eastern portion of the range (10–12 m [16]; 9.6–11.2 m [1]). Diet of the southern Madagascar whales was also distinctively different from other southern African Bryde’s whales, being composed entirely of euphausiids with equal proportions of *E. diomedeae* and *E. recurva* [17,39], whereas the inshore whales taken off Durban (Donkergat station) fed almost exclusively on shoaling fishes [39]. There were no descriptions of external appearance or scarring reported for the southern Madagascar whales [39]. Best [39] also described sightings of Bryde’s whales and ‘like-Bryde’s’ whales to the south of Madagascar during surveys by whaling sighting vessels during December 1996. Best [39] concluded that these animals represented a third population in southern Africa, distinct from the South African Inshore Stock and the Southeast Atlantic Offshore Stock. Given the observations on similar size and diet, it seems feasible that these could represent *B. omurai* with the implication that the species may range further than suggested above, or engage in seasonal migration. In discussion with P.B. Best about our northwest Madagascar sightings prior to obtaining genetic confirmation of species-ID, upon inspecting the photographs and underwater video from 2013 (figure 3, first row) Best (12 December 2013, personal communication) indicated that he was unfamiliar with this whale and that he did not think it was a Bryde’s whale. Therefore, as Best personally observed the whales from the south of Madagascar, then they probably represent not *B. omurai* but a separate population of Bryde’s whales. However, given the similarities noted, if genetic material or photographs from the scientific whaling operations exist, it would be important to confirm species identity and exclude *B. omurai* as a candidate. Best [39] (citing [47]) also reports the catch of an ‘abnormal’ Bryde’s whale landed at the Durban whaling station on the southeastern coast of South Africa with asymmetrical coloration similar to a *B. physalus*, many cookie cutter scars, and a length of 14.95 m; despite the pigmentation asymmetry, the other descriptions do not match that of *B. omurai* as observed in Madagascar. Therefore, it seems unlikely that this specimen belongs to the Madagascar population of *B. omurai*.

### 4.3 Extent of range

The existence of a population of *B. omurai* off Madagascar, as well as other recent reports of the species, dramatically revises the understanding of this species global range. In an effort to present an up-to-date overview of all existing knowledge, we have compiled all accounts (to the best of our knowledge) and presented it as electronic supplementary material (figure S4, table S2), including the peer-reviewed literature, scientific reports and web-based blog and media accounts. The main concentration of accounts remains in the western tropical/subtropical Pacific, with the most reliable eastern range extent being New Caledonia [23] at longitude 167° E (electronic supplementary material, table S2, figure S4), with photographic evidence corroborated by one of us (S.C.) as strongly resembling Madagascan individuals’ external appearance. The most northern extent reported remains the holotype specimen from the Sea of Japan (34°21′ N, 130°50′ E) [1]. The most southern extent is the report of a stranding identified by skull morphology [21] in Gulf St Vincent, South Australia, at latitude 34°37.6′ S (electronic supplementary material, table S2, figure S4). Although these northern and southernmost records are at comparable latitudes, until more detailed information is available on the status and range of populations, it is possible that they may represent extralimital accounts. Recent accounts in the literature [19] and media of genetic and photographic evidence off the northwest coast of Australia (electronic supplementary material, table S2, figure S4) suggest that the species may be more common than previously known in southeast Indian Ocean waters off northern Australia. In the northeast Indian Ocean, there are accounts off the west coast of Thailand [7,22] and good video evidence in the eastern Andaman Sea (electronic supplementary material, table S2, figure S4); accounts from the Gulf of Thailand [22] and Malaysia [9] indicate that the species occurs on both sides of the Malay Peninsula. It is noteworthy that relatively extensive sampling of Bryde’s whale populations in the North Indian Ocean, including the Arabian Sea, Bay of Bengal, the Maldives and south of Java, did not reveal evidence of *B. omurai* in the genetic samples [3]. This infers that the species distribution is discontinuous, and the Madagascar population in the southwest Indian Ocean may be relatively isolated from the eastern populations; however, it is conceivable that other populations have gone undetected, as the Cocos Islands account [1] indicates that populations occur around oceanic island groups in the South Indian Ocean. However, the Cocos Islands records may be anomalous as they were reported collected 110–275 km offshore the nearest island in more than 4000 m depth (as determined by positions reported in [1]). Probably the most surprising account is a recent report of a genetically identified stranding off Mauritania in the North Atlantic Ocean at 16°32.5′ N, 16°27′ W [20]; the stranded individual was of neonate size, and thus the authors infer that it is more likely indicative of a local population as opposed to an extralimital accidental occurrence. If a North Atlantic population is further validated, then it suggests the range of the species is more global in character than Indo-Pacific, as would be inferred from a range extension to Madagascar alone.

### 4.4 Reproductive ecology and acoustic behaviour

The presence of mothers with young calves suggests that our study site probably represents breeding habitat where females give birth, and moreover that there appears to be no temporal or spatial separation of breeding and feeding in *B. omurai*. This is distinctly different from large migratory baleen whales but is congruent with what is known about at least some populations of Bryde’s whales, such as the South African Inshore Stock [39] and the Gulf of Mexico population [4,48,49]. Regarding seasonal timing of parturition, Yamada [16] reports a neonate of 3 m in length in late August in Miyazaki Prefecture, southern Japan; our observations of young calves in August and in November are both congruent with this report, and suggest that birthing may occur over a protracted period, but more data are needed to determine whether it is seasonal or year round. Reproduction in inshore Bryde’s whales off South Africa appeared to be seasonally unrestricted, and in offshore Bryde’s whales protracted throughout the year with seasonal peaks [39]. The dramatic difference in encounter rates of mother–calf pairs between 2013 and 2014 in Madagascar is perplexing, raising more questions about reproduction and habitat usage for the species and only emphasizes the need for long-term study and assessment of seasonal variation in sighting characteristics, distribution and behaviour.

The vocalizations that we report here and attribute to *B. omurai* have the following key characteristics: (i) they are stereotyped in structure, with a distinct and consistent recognizable pattern; (ii) they are uttered in consistent rhythmic series, for extended periods of time; and (iii) at times there are choruses of multiple individuals phonating within audible range of the sensor, and therefore, within range of one another. These are the key characteristics of baleen whale song (most elaborate in humpback whales [50]), and thus we propose that this is probably a sexually selected song display similar to the simple displays of other *Balaenoptera* such as *B. physalus* and *Balaenoptera musculus* [51–53]. If this hypothesis is accurate, then we predict that it is a male-limited trait, will display individual variation, and studies of breeding behaviour will reveal it is an inter-sexual and/or intra-sexual signal and integral part of the species mating system. That the sightings of *B. omurai* often occurred in loose aggregations of individuals is probably of consequence to this discussion. It is not possible with our current data to make inferences as to whether the aggregating of individuals is related to exploitation of a common patchy food resource, or related to social and reproductive behaviour, or potentially both. Several observations suggest that it may be at least in part related to breeding behaviour. The female that was re-sighted across years was sighted in the first year within an aggregation of several individuals, and then in the following year with a calf; it is therefore possible that a female’s involvement in an aggregation is functional in courtship. On at least one occasion (described above) an aggregation of several individuals was observed for hours without straying out of a small area, and involved one of the rare observations of direct social interaction of two individuals and breaching. We speculate that these aggregations may represent groups of animals engaged in courtship behaviour, possibly involving singing males interacting in a lek-like mating system, as has been proposed for humpback whales [54,55]. If the population of *B. omurai* off Madagascar is in fact small and resident, then focused behavioural studies on acoustic and individual behaviour, coupled with molecular ecology studies on paternity, may be feasible and reveal a substantive understanding of the reproductive ecology of the species.

### 4.5 Evolutionary and ecological questions

Many interesting evolutionary questions remain regarding *B. omurai*. The great similarity in pigmentation with *B. physalus* would suggest that the complex character is an ancestral sympleisiomorphy (it is not a synapomorphy by definition, as pointed out by Sasaki *et al.* [2]). A phylogenetic reconstruction that would make this a parsimonious explanation would place *B. physalus* nested outside and basal to the Bryde’s/sei/Omura’s whale clade, and therefore inferring a single evolution and then a single consequent loss of the complex set of characters. However, in most phylogenetic hypotheses there are other species grouped with *B. physalus* (*Megaptera novaeangliae* or *B. musculus*) or nested in between *B. physalus* and *B. omurai* (primarily *B. musculus*) [1,2,4,6]; given the distance of the two taxa and these complex branching patterns, a common ancestral hypothesis would require multiple losses of the character across extant taxa. If not due to common ancestry, the convergent evolution of such precise similarity in pigmentation (with asymmetry, white lower right jaw, light blaze and chevron, and dark eye and ear stripes) in two species that are ecologically so different seems equally non-parsimonious. This infers that there may still be considerable work needed to resolve the phylogenetic relationships among mysticetes; the difficulty may in part be owing to the lack of a rigorous multi-gene phylogeny incorporating both nuclear and mitochondrial markers which may reveal a ‘species-tree’ that is different from current ‘gene-trees’. Other explanations, such as developmental constraints on phenotypic expression of pigmentation patterns, or reticulation within the phylogeny and introgression of genotypes and associated phenotypes from one distantly related taxa to another, seem less parsimonious and more speculative than a common sympleisiomorphic evolutionary origin of the trait. Irrespective of speculation, investigation of such similar pigmentation pattern in two taxa that are as phylogenetically distinct and ecologically different as reported is warranted and should prove a fruitful endeavour.

As noted by Yamada [16], affiliation of *B. omurai* with Bryde’s whales is misleading, particularly to non-specialists. Despite Wada *et al.* [1] and Sasaki *et al.* [2] establishing the phylogenetic distinctiveness, recent papers at times refer to a ‘Bryde’s whale complex’ that includes *B. edeni* ssp. and *B. omurai*, or make reference to *B. omurai* in context of diagnostic molecular Characteristic Attribute analysis (CA) with *B. edeni* ssp. [3,4]; however, this grouping would have to include the sei whale, *B. borealis*, to be phylogenetically accurate and meaningful in a CA analysis. Given the sister relationship and relatively shallow divergence time of *B. edeni* and *B. borealis* as compared to the more ancient divergence of *B. omurai*, the original confusion of *B. omurai* with *B. edeni* appears to be more related to similarity in size and general ecology than due to a close phylogenetic relationship. Therefore, we recommend (following on Yamada [16]) that the use of the term ‘Bryde’s whale complex’ be used only for the currently putative subspecies of *B. edeni* until those relationships and nomenclature are resolved. Moreover, from an evolutionary ecology perspective, if both *B. edeni* and *B. omurai* are non-migratory, tropical/subtropical species as suspected, then they either represent convergent evolution of similar ecological characters in two distinct lineages, or alternatively, a common ancestral life-history pattern for the clade and the reversal of the trait in *B. borealis*. In either case, a non-migratory, tropical/subtropical ecology is rare among mysticetes and in the minority among Balaenopteridae. Therefore, comparative studies of populations of *B. edeni* and *B. omurai* will provide insights into the ecological pressures that influence the evolution of this life history, and adaptations that arise in response to an exclusively low-latitude range (e.g. small size relative to congeners is apparently one candidate adaptation).

### 4.6 Conservation issues

*Balaenoptera omurai* is assessed as ‘data deficient’ on the IUCN Red List [56]. Range-wide there are no reliable population size estimates, trends or evaluation of conservation threats. In regards to directed takes, it is known to have been taken in Japanese research whaling in small numbers [1,5], as well as shore-based Philippine artisanal whaling in the Bohol Sea [8,12] in numbers which may have been substantial in comparison to population size. Given the poor information on range, it is possible that the species may have been subjected to commercial whaling and gone unnoticed [56], but this is currently unclear. By-catch (entanglement) in local fisheries has been reported at least in Songkhla, Thailand [22], and given an apparent shallow-water habitat preference, it is likely that the species is vulnerable to by-catch throughout its range. Without more information on population ranges and abundance, it is difficult to assess impacts of documented and suspected takes, and it is recommended that more effort be made to assess populations throughout its global range.

The information presented here suggests that the Omura’s whale population (subpopulation in IUCN terms) in northwest Madagascar may be small, resident and isolated with low genetic diversity, further emphasizing the need to understand its biology, distribution and potential conservation concerns. Based on the prevalence of the low frequency vocalizations of this species, it appears that these whales are highly vocal and acoustic communication is probably a critical component of social and breeding behaviour. In addition, their vocal nature and low frequency bandwidth of their vocalizations make them vulnerable to noise associated with anthropogenic activities, particularly seismic exploration associated with hydrocarbon exploration and production (E&P). These powerful signals would present a strong negative stimulus in a bandwidth for which they are highly attuned, and may mask key vocalizations critical for life functions. A growing body of evidence indicates that communication of baleen whales is impacted by seismic surveys, including the disruption of song displays [57–61]. The current presence and planned future expansion of hydrocarbon E&P (http://www.oilandgastechnology.net/upstream-news/sterling-extends-exploration-licence-madagascar-offshore-blocks; http://www.puravidaenergy.com.au/operations/madagascar/) within the documented range of the Madagascar Omura’s whale population is of significant conservation concern to this species and other acoustically sensitive species of the northwest region of Madagascar. The Ankivonjy and Ankarea MPAs that partially overlap the Omura’s whale population habitat were afforded permanent status in 2015, and may confer some degree of protection, but only within relatively small core areas where industry activities are restricted. Therefore, this subpopulation of Omura’s whales off the northwest coast of Madagascar should be assessed for inclusion on the IUCN Red List, and further work is recommended to establish its conservation status.

## Supplementary Material

Cerchio et al - Omuras Suppl Info Revision1 FINAL.docx Supplementary figures and tables, including Genbank sequences used in analysis, photographs displaying individual variation in external appearance, a map of all currently known locations of Omura's whale accounts, and a review of existing accounts of Omura's whales globally.

## Data accessibility

Sequences have been deposited in GenBank accession no. KT582064; and the alignment used in phylogenetic analysis made available at http://commons.nmu.edu/facwork_datasets/1/.
