# Supplementary material for: Omura’s whales (Balaenoptera omurai) off northwest Madagascar: ecology, behaviour and conservation needs
Source: R Soc Open Sci. 2015 Oct 14;2(10):150301. doi: 10.1098/rsos.150301 (PMC4632516; doi:10.1098/rsos.150301)
Supplement: Cerchio et al - Omuras Suppl Info Revision1 FINAL.docx Supplementary figures and tables, including Genbank sequences used in analysis, photographs displaying individual variation in external appearance, a map of all currently known locations of Omura's whale accounts, and a review of existing accoun [file rsos150301supp1.pdf]

Omura's whales (*Balaenoptera omurai*) off northwest Madagascar: ecology, behavior and conservation needs

SALVATORE CERCHIO[1,2], BORIS ANDRIANANTENAINA[3], ALEC LINDSAY[4], MELINDA REKDAHL[5], NORBERT ANDRIANARIVELO[3] AND TAHINA RASOLOARIJAO[3]

[1]*New England Aquarium, Boston, MA, USA*
[2]*Woods Hole Oceanographic Institution, Woods Hole, MA, USA*
[3]*Institut Halieutique et des Sciences Marines, Universite de Toliara, Toliara, Madagascar*
[4]*Northern Michigan University, Biology Department, Marquette, MI, USA*
[5]*Wildlife Conservation Society, Ocean Giants Program, Bronx, NY, USA*

Correspondence: scerchio@whoi.edu

SUPPLEMENTAL INFORMATION

Table S1. Mysticete species sequences used in this study.

Genbank	Specimen	English Name	Figure 2 label
KT582064	*Balaenoptera omurai*	Omura's whale	B_omurai (Madagascar)
AB116095	*Balaenoptera omurai*	Omura's whale	B_omurai (Japan1)
AB116096	*Balaenoptera omurai*	Omura's whale	B_omurai (Solomon Is.)
AB116097	*Balaenoptera omurai*	Omura's whale	B_omurai (Cocos Is.)
AB201257	*Balaenoptera omurai*	Omura's whale	B_omurai (Japan2)
AF398372**	*Balaenoptera edeni (sic)*	Omura's whale	B_omurai (East China Sea)
AP006470	*Balaenoptera borealis*	Sei whale	B_borealis1
X72195	*Balaenoptera borealis*	Sei whale	B_borealis2
AB116098	*Balaenoptera brydei*	Bryde's whale	B_brydei4
EU030282	*Balaenoptera brydei*	Bryde's whale	B_brydei1
AB201259	*Balaenoptera brydei*	Bryde's whale	B_brydei3
AP006469	*Balaenoptera brydei*	Bryde's whale	B_brydei2
X72196	*Balaenoptera edeni*	Eden's whale	B_edeni1
GU085096	*Balaenoptera edeni*	Eden's whale	B_edeni2
GU085098	*Balaenoptera edeni*	Eden's whale	B_edeni3
KC572744	*Balaenoptera physalus*	Fin whale	B_physalus2
KC572810	*Balaenoptera physalus*	Fin whale	B_physalus3
KC572821	*Balaenoptera physalus*	Fin whale	B_physalus1
KC572824	*Balaenoptera physalus*	Fin whale	B_physalus4
AP006466	*Balaenoptera bonaerensis*	Antarctic minke whale	B_bonaerensis
X72204	*Balaenoptera musculus*	Blue whale	B_musculus
AP006473	*Eubalaena australis*	Southern right whale	Eubalaena_australis

**Rosel and Wilcox [1] notes that this sequence, from Yang *et al.* [2] before the identification of *B. omurai*, is mis-identified on Genbank as *B. edeni.*

Figure S1. Variation among individuals in blaze pigmentation pattern of *Balaenoptera omurai* in northwest Madagascar.

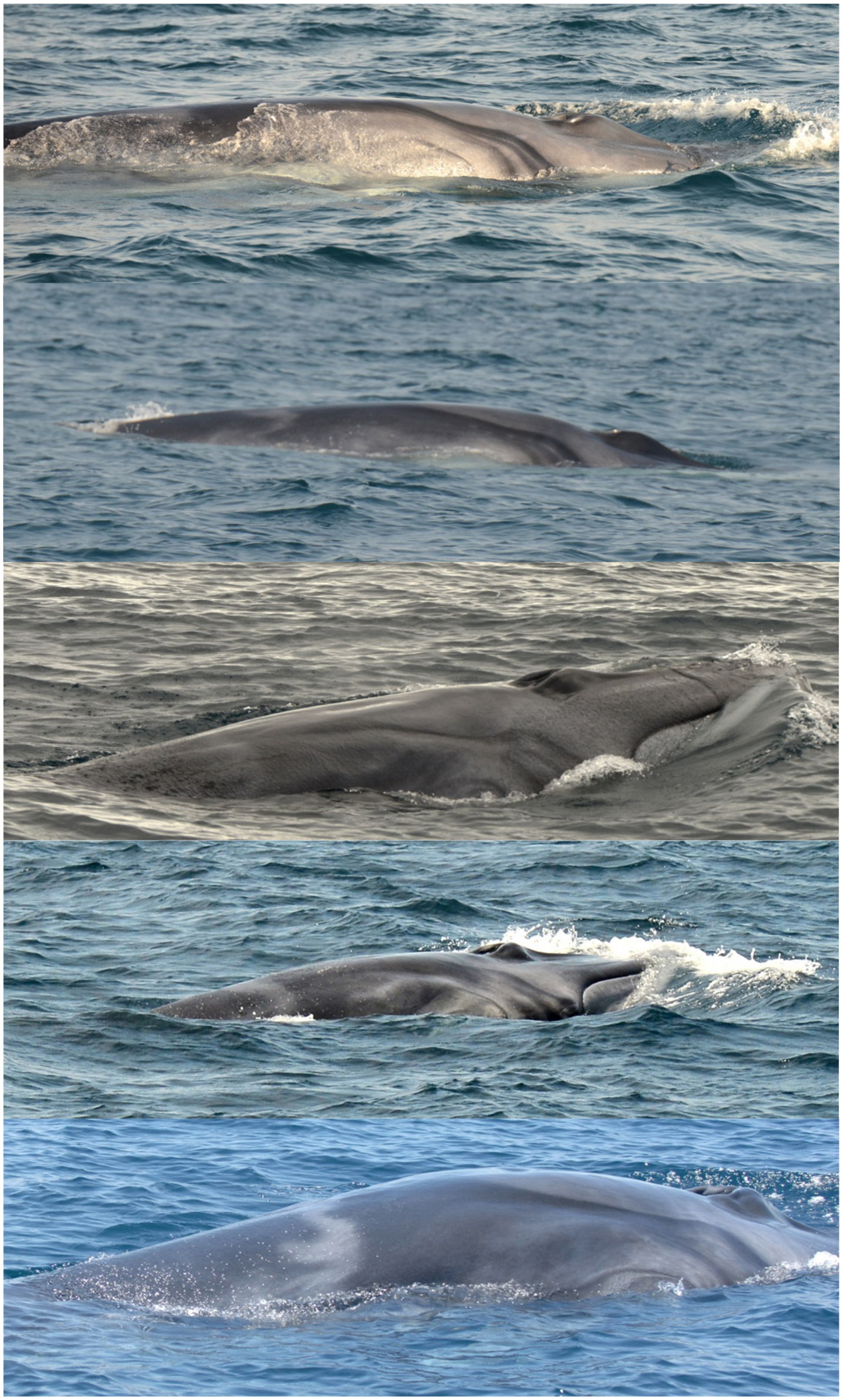

Figure S2. Variation among individuals in chevron pigmentation pattern of *Balaenoptera omurai* in northwest Madagascar.

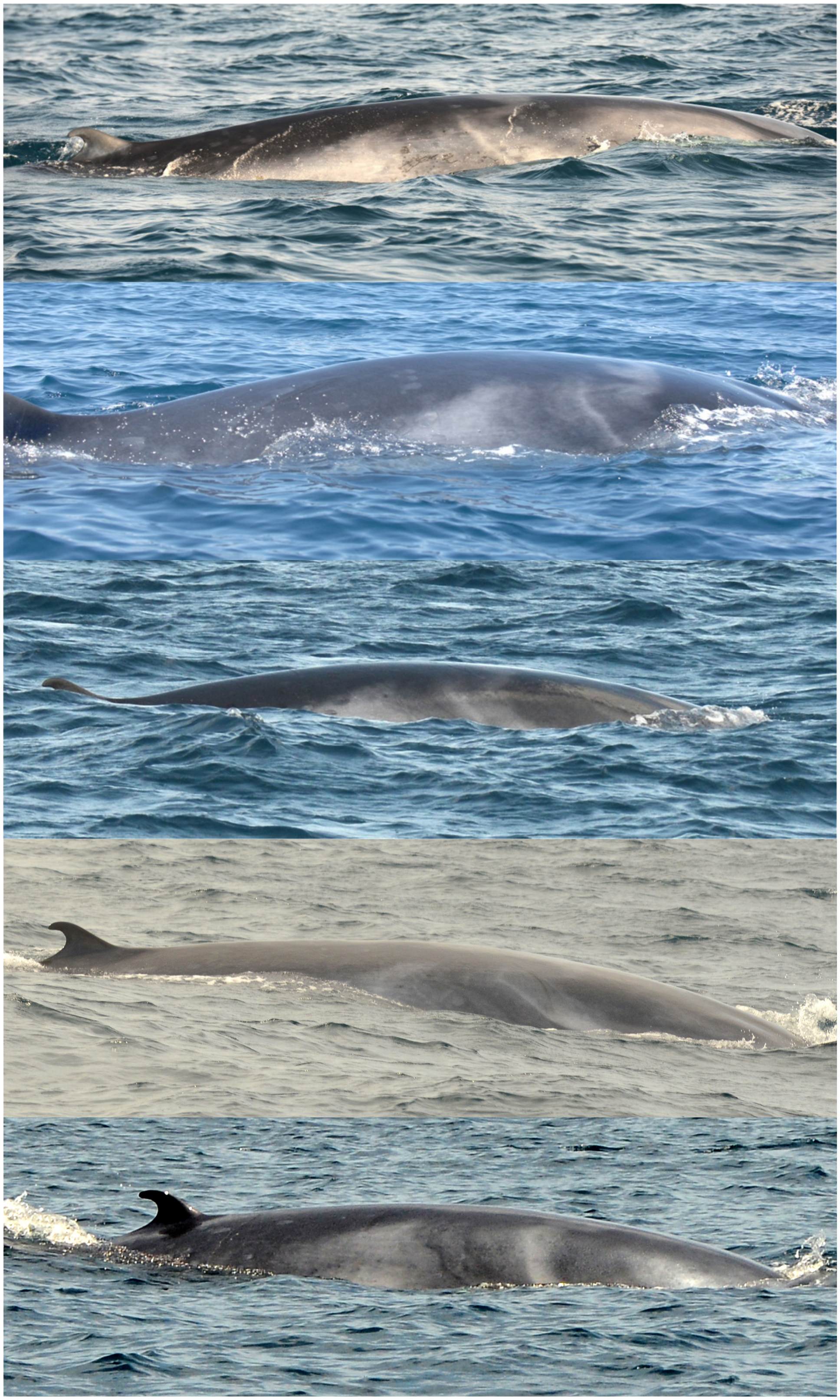

Figure S3. Variation among individuals in dorsal fin shape of *Balaenoptera omurai* in northwest Madagascar.

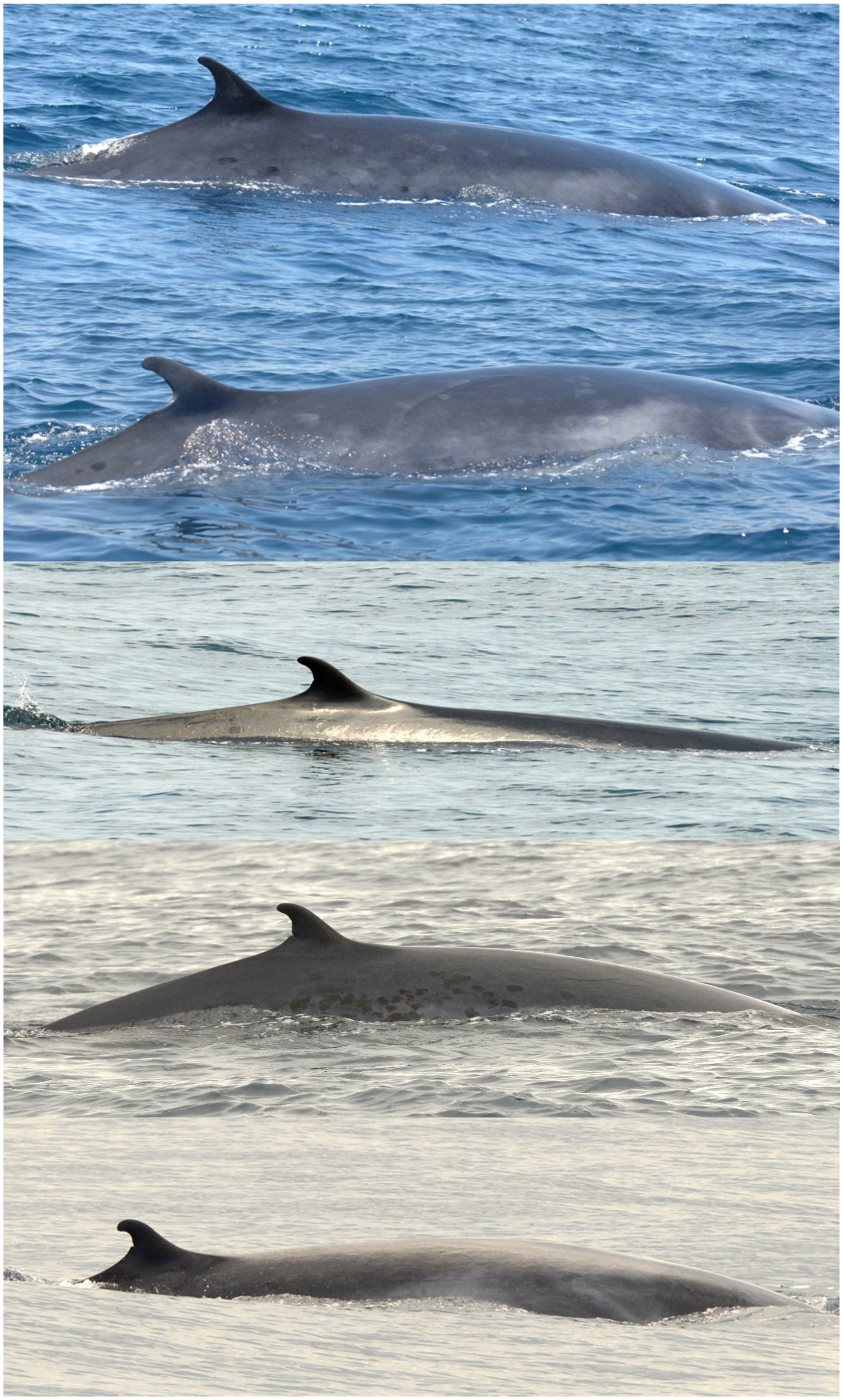

Figure S4. Currently reported documentation of Omura's whales globally, based on peer-reviewed literature, scientific reports, and web-based information as detailed in Table S2. Each marker refers to a single account, but may involve multiple individuals. Markers are coded as the type of documentation, and when multiple types are reported in a single account, the highest priority type is indicated in order of: molecular genetic species identification, skull morphology assessment, and photographic or video documentation.

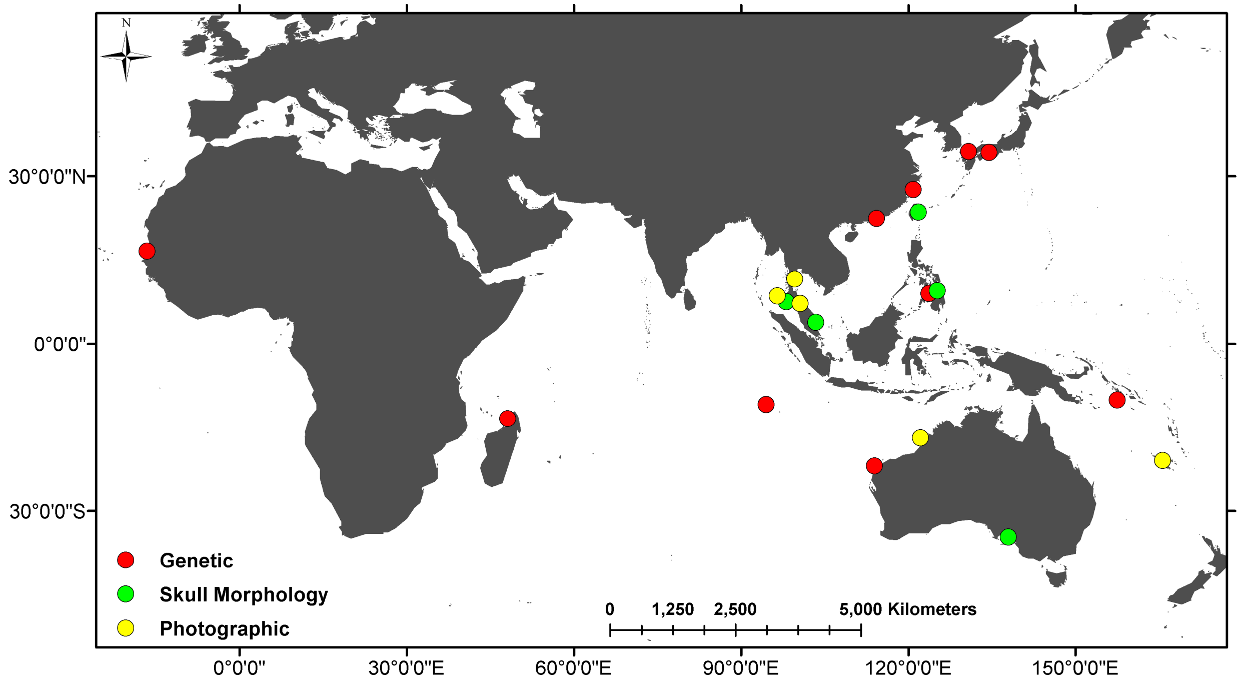

Table S2. Summary review of all accounts of Omura's whales currently available, globally, based on peer-reviewed literature, scientific reports, and web-based information. Species identification is based on molecular genetic evidence, skull morphology, or photographic evidence (corroborated by S.C.). At the bottom of the table, two web-based photographic/video accounts reported as potential Omura's whales are included that the principle author (S.C.) could not corroborate due to external appearance not being congruent with Madagascar population.

Acct#	Museum/Ref #	Citation/Source	Specimen Source	Specimen Type	GenBank #	GenBank Sequence Type	Country
JA-01	NSMT-M32505	Wada et al 2003 [3]; Sasaki et al 2006 [4]	Stranding	Tissue (genetic)	AB116095; AB201256	mtDNA D-Loop; mtDNA genome	Japan
JA-02	NSMT-M32992	Sasaki et al 2006 [4]	Stranding	Tissue (genetic)	AB201257	mtDNA genome	Japan
CH-01	NJNU0379	Yang et al 2002 [2]	Stranding	Tissue (genetic)	AF398372	mtDNA D-loop	China
SI-01	NRIFSF1	Wada et al 2003 [3]; Yoshida & Kato 1999 [5]	Research Whaling	Tissue (genetic)	AB116096; AF146389	mtDNA D-loop	Soloman Islands
SI-02	NRIFSF2	Wada et al 2003 [3]; Yoshida & Kato 1999 [5]	Research Whaling	Tissue (genetic)	not uploaded	mtDNA D-loop	Soloman Islands
SI-03	NRIFSF3	Wada et al 2003 [3]; Yoshida & Kato 1999 [5]	Research Whaling	Tissue (genetic)	not uploaded	mtDNA D-loop	Soloman Islands
SI-04	NRIFSF4	Wada et al 2003 [3]; Yoshida & Kato 1999 [5]	Research Whaling	Tissue (genetic)	not uploaded	mtDNA D-loop	Soloman Islands
SI-05	NRIFSF5	Wada et al 2003 [3]; Yoshida & Kato 1999 [5]	Research Whaling	Tissue (genetic)	not uploaded	mtDNA D-loop	Soloman Islands
SI-06	NRIFSF6	Wada et al 2003 [3]	Research Whaling	Tissue (genetic)	not sequenced?	n/a	Soloman Islands
CI-01	NRIFSF7	Wada et al 2003 [3]	Research Whaling	Tissue (genetic)	AB116097	mtDNA D-loop	Cocos (Keeling) Islands
CI-02	NRIFSF8	Wada et al 2003 [3]	Research Whaling	Tissue (genetic)	not sequenced?	n/a	Cocos (Keeling) Islands
TW-01	NMNS 1004	Yamada, Chou et al 2006 [6]	Stranding	incomplete skeleton	n/a	n/a	Taiwan
TW-02	NMNS 1931 (TN9707)	Yamada, Chou et al 2006 [6]	Stranding	whole skeleton and baleen plates	n/a	n/a	Taiwan
TW-03	NMNS 2463 (ML9702)	Yamada, Chou et al 2006 [6]	Stranding	whole skeleton and baleen plates	n/a	n/a	Taiwan
TW-04	TP9702	Yamada, Chou et al 2006 [6]	Stranding	Incomplete skeleton	n/a	n/a	Taiwan
TW-05	NMNS6619 (TY9802)	Yamada, Chou et al 2006 [6]	Stranding	whole skeleton and baleen plates	n/a	n/a	Taiwan
TW-06	HL20040127	Yamada, Chou et al 2006 [6]	Stranding	whole skeleton and soft tissues	n/a	n/a	Taiwan
TW-07	PCAQ01	Yamada, Chou et al 2006 [6]	Stranding	Incomplete skeleton	n/a	n/a	Taiwan
HK-01	?	http://hkmarinelife.hk/2014/03/31/whale-carcass-stranded-near-taipo/ http://hkmarinelife.hk/2014/12/23/taipo-whale-identified-as-recently-discovered-omuras-whale/	Stranding	Tissue (genetic); whole skeleton	?	?	Hong Kong
TL-01	PMBC11621 (End013)	Yamada, Chou et al 2006 [6]	Stranding	whole skeleton	n/a	n/a	Thailand
TL-02	PMBC11630 (End030)	Yamada, Chou et al 2006 [6]	Stranding	whole skeleton	n/a	n/a	Thailand
TL-03	End156	Yamada, Chou et al 2006 [6]	Stranding	whole skeleton	n/a	n/a	Thailand
TL-04	n/a	Adulyanukosol et al 2012 [7]	Stranding	Photographs	n/a	n/a	Thailand
TL-05	n/a	Adulyanukosol et al 2012 [7]	Live Sighting	Report	n/a	n/a	Thailand
TL-06	n/a	Adulyanukosol et al 2012 [7]	Stranding	Report	n/a	n/a	Thailand
TL-07	n/a	Adulyanukosol et al 2012 [7]	Stranding	Report	n/a	n/a	Thailand
TL-08	n/a	https://www.youtube.com/watch?v=3JSrl2bd0-g	Live Sighting	video	n/a	n/a	Thailand
PH-01	SUML0142	Yamada et al 2008 [8]	Philippines Whaling	Partial Skull	n/a	n/a	Philippines

Table S2. Continued.

Acct#	Location	Latitude	Longitude	Body length (m)	Sex	Physical maturity	Date of Account	Comment
JA-01	Sea of Japan - Tsunoshima Island	34º 21’ N	130º 50’ E	11.03	F	Adult	11 September 1998	Holotype specimen
JA-02	Inland Sea - Shikoku, Kagawa Prefecture	?	?	?	?	?	4 June 2002	
CH-01	Rui'an City, Zhejiang, China	?	?	?	?	?	?	Labeled B.edeni on GenBank
SI-01	Soloman Islands	10º 03’S	157º 29’ E	11.5	F	Adult	24 October 1976	
SI-02	Soloman Islands	9º 53’ S	157º 37’ E	9.6	M	Adult	24 October 1976	
SI-03	Soloman Islands	9º 57’ S	157º 41’ E	11.2	F	Adult	24 October 1976	
SI-04	Soloman Islands	9º 49’ S	157º 29’ E	10	M	Adult	24 October 1976	
SI-05	Soloman Islands	10º 07’ S	157º 51’ E	10.3	F	Adult	24 October 1976	
SI-06	Soloman Islands	10º 17’ S	157º 56’ E	9.6	M	Adult	24 October 1976	
CI-01	Cocos (Keeling) Islands	10º 51’ S	97º 02’ E	10.4	F	Adult	15 November 1978	
CI-02	Cocos (Keeling) Islands	10º 53’ S	94º 29’ E	10.1	F	Immature	17 November 1978	
TW-01	Pingtung County	?	?	?	?	?	5 November 1990	
TW-02	Tainan County	?	?	5.54	M	?	11 February 1997	
TW-03	Miaoli County	?	?	5.13	M	?	5 March 1997	
TW-04	Taipei County	?	?	8 to 9	M	?	8 May 1997	
TW-05	Taoyuan County	?	?	6.54	M	?	1 December 1998	
TW-06	Hualien County	?	?	5.95	F	?	27 January 2004	
TW-07	?	?	?	?	?	?	?	
HK-01	Hung Shek Mun, Plover Cove Country Park, Tai Po	22°30'15"N	114°17'51"E	10.8	F?	Mature	31 March 2014	Genetic verification of speceis identity and stranding data provided by R.L.Brownell Jr & R. Brown, pers. comm. 19 May 2015
TL-01	Phuket Province	?	?	7	?	?	15 September 1983	
TL-02	Phuket Province	?	?	10	M	?	12 June 1995	
TL-03	Phang-nga Province	?	?	4.3	F	?	1 December 1999	
TL-04	Songkhla Pronvince	?	?	4.4	?	Juvenile	31 May 2011	Bycaught calf pictured on pg 26+102 of book
TL-05	Phuket Island / Racha Noi-	?	?	Unk	Unk	n/a	?	Visual sighting noted on pg 27 of book
TL-06	Bang Pat, Phang-nga Province	?	?	4.3	?	Juvenile	?	Stranded calf noted on pg 27 of book; possibly = TL03
TL-07	Thap Sakae, Prachuap Khiri	?	?	3.87	?	Juvenile	?	Stranded calf noted on pg 27 of book
TL-08	Tachai Pinnacle, National Park Similan Islands	?	?	Unk	Unk	n/a	9 February 2013	Visual ID corroborated by S.Cerchio
PH-01	Bohol Sea	Unk	Unk	Unk	Unk	?	1980's	

Table S2. Continued.

Acct#	Museum/Ref #	Citation/Source	Specimen Source	Specimen Type	GenBank #	GenBank Sequence Type	Country
PH-02	SUML0144	Yamada et al 2008 [8]	Philippines Whaling	Partial Skull	n/a	n/a	Philippines
PH-03	SUML0145	Yamada et al 2008 [8]	Philippines Whaling	Partial Skull	n/a	n/a	Philippines
PH-04	SUML0148	Yamada et al 2008 [8]	Philippines Whaling	Partial Skull	n/a	n/a	Philippines
PH-05	SUML0149	Yamada et al 2008 [8]	Philippines Whaling	Partial Skull	n/a	n/a	Philippines
PH-06	SUML0150	Yamada et al 2008 [8]	Philippines Whaling	Partial Skull	n/a	n/a	Philippines
PH-07	SUML0151	Yamada et al 2008 [8]	Philippines Whaling	Partial Skull	n/a	n/a	Philippines
PH-08	SUML0152	Yamada et al 2008 [8]	Philippines Whaling	Partial Skull	n/a	n/a	Philippines
PH-09	SUML0153	Yamada et al 2008 [8]	Philippines Whaling	Partial Skull	n/a	n/a	Philippines
PH-10	SUML0156	Yamada et al 2008 [8]	Philippines Whaling	Partial Skull	n/a	n/a	Philippines
PH-11	SUML0157	Yamada et al 2008 [8]	Philippines Whaling	Partial Skull	n/a	n/a	Philippines
PH-12	SUML0208	Yamada et al 2008 [8]	Philippines Whaling	Partial Skull	n/a	n/a	Philippines
PH-13	SUML0209	Yamada et al 2008 [8]	Philippines Whaling	Partial Skull	n/a	n/a	Philippines
PH-14	SUML0210	Yamada et al 2008 [8]	Philippines Whaling	Partial Skull	n/a	n/a	Philippines
PH-15	SUML0211	Yamada et al 2008 [8]	Philippines Whaling	Partial Skull	n/a	n/a	Philippines
PH-16	SUML0212	Yamada et al 2008 [8]	Philippines Whaling	Partial Skull	n/a	n/a	Philippines
PH-17	SUML0213	Yamada et al 2008 [8]	Philippines Whaling	Partial Skull	n/a	n/a	Philippines
PH-18	SUML0215	Yamada et al 2008 [8]	Philippines Whaling	Partial Skull	n/a	n/a	Philippines
PH-19	SUML0217	Yamada et al 2008 [8]	Philippines Whaling	Partial Skull	n/a	n/a	Philippines
PH-20	SUML0218	Yamada et al 2008 [8]	Philippines Whaling	Partial Skull	n/a	n/a	Philippines
PH-21	SUML0219	Yamada et al 2008 [8]	Philippines Whaling	Partial Skull	n/a	n/a	Philippines
PH-22	SUML0220	Yamada et al 2008 [8]	Philippines Whaling	Partial Skull	n/a	n/a	Philippines
PH-23	SUML0221	Yamada et al 2008 [8]	Philippines Whaling	Partial Skull	n/a	n/a	Philippines
PH-24	SUML0475	Yamada et al 2008 [8]	Philippines Whaling	Partial Skull	n/a	n/a	Philippines
ML-01	?	Ponnampalam et al 2012 [9]	Stranding	Skull	n/a	n/a	Malaysia
NC-01	n/a	Garrigue and Poupon 2013 [10]	Live Sighting	Photographs	n/a	n/a	New Caledonia
NC-02	n/a	https://mersociety.wordpress.com/2012/11/07/extraordinarily-rare-whale-sighting-omuras-whale-balaenoptera-omurai/	Live Sighting	Photographs	n/a	n/a	New Caledonia
NC-03	n/a	Van Canneyt et al 2015 [11]	Live Sighting	Photographs	n/a	n/a	New Caledonia
AU-01	South Australian Museum M21245	Yamada, Kemper et al 2006 [12]	Stranding	whole skeleton	n/a	n/a	Australia
AU-02	n/a	http://www.pbase.com/wildlifeimages/omuras_whale	Live Sighting	Photographs	n/a	n/a	Australia
AU-03	?	Ottewell et al In press [13]	Stranding	Tissue (genetic), photographs	?	?	Australia
MU-01	MauBs	Jung et al 2015 [14]	Stranding	Tissue (genetic)	KM233837; KM233838; KM233839	mtDNA Dloop; Cytb;Cox1	Mauritania
PH-24	n/a	https://www.youtube.com/watch?v=Sw3BbZvx63A https://www.youtube.com/watch?v=GGIxDMAron0	live stranding	Video	n/a	n/a	Philippines
CI-01	n/a	http://www.whaleresearch.org/ourwork/mystery_whale.php	Live Sighting	Photographs	n/a	n/a	Cook Islands

Table S2. Continued.

Acct#	Location	Latitude	Longitude	Body length (m)	Sex	Physical maturity	Date of Account	Comment
PH-02	Bohol Sea	Unk	Unk	Unk	Unk	?	1980's	
PH-03	Bohol Sea	Unk	Unk	Unk	Unk	?	1980's	
PH-04	Bohol Sea	Unk	Unk	Unk	Unk	?	1980's	
PH-05	Bohol Sea	Unk	Unk	Unk	Unk	?	1980's	
PH-06	Bohol Sea	Unk	Unk	Unk	Unk	?	1980's	
PH-07	Bohol Sea	Unk	Unk	Unk	Unk	?	1980's	
PH-08	Bohol Sea	Unk	Unk	Unk	Unk	?	1980's	
PH-09	Bohol Sea	Unk	Unk	Unk	Unk	?	1980's	
PH-10	Bohol Sea	Unk	Unk	Unk	Unk	?	1980's	
PH-11	Bohol Sea	Unk	Unk	Unk	Unk	?	1980's	
PH-12	Bohol Sea	Unk	Unk	Unk	Unk	?	1980's	
PH-13	Bohol Sea	Unk	Unk	Unk	Unk	?	1980's	
PH-14	Bohol Sea	Unk	Unk	Unk	Unk	?	1980's	
PH-15	Bohol Sea	Unk	Unk	Unk	Unk	?	1980's	
PH-16	Bohol Sea	Unk	Unk	Unk	Unk	?	1980's	
PH-17	Bohol Sea	Unk	Unk	Unk	Unk	?	1980's	
PH-18	Bohol Sea	Unk	Unk	Unk	Unk	?	1980's	
PH-19	Bohol Sea	Unk	Unk	Unk	Unk	?	1980's	
PH-20	Bohol Sea	Unk	Unk	Unk	Unk	?	1980's	
PH-21	Bohol Sea	Unk	Unk	Unk	Unk	?	1980's	
PH-22	Bohol Sea	Unk	Unk	Unk	Unk	?	1980's	
PH-23	Bohol Sea	Unk	Unk	Unk	Unk	?	1980's	
PH-24	Bohol Sea	Unk	Unk	Unk	Unk	?	1980's	
ML-01	Pahang	?	?	?	?	?	2008	Spec ID by T.Yamada, pers comm to citation author
NC-01	Southern New Caledonia	22º 30.787' S	166º 47.808' E	Unk	Unk	n/a	17 November 2010	Sightings reported from 1997 to 2014; data provided by C. Garrigue, pers. comm. 15 Sept 2015
NC-02	Southern New Caledonia	22º 30.0' S	166º 59.6' E	Unk	Unk	n/a	14 May 2012	Visual ID corroborated by S. Cerchio
NC-03	Eastern New Caledonia	20º 48.759' S	165º 26.517' E	Unk	Unk	n/a	13 November 2014	Three visual sightings reported during aerial surveys
AU-01	Black Point, Yorke Peninsula, Gulf St Vincent, South Australia	34º 37.566' S	137º 56.334' E	10.2	Unk	Subadult	17 January 2000	Data on stranding provided by the South Australian Museum, C. Kemper, Pers. Comm. 1 Sept 2015
AU-02	north of the Lacapede Islands	?	?	Unk	Unk	n/a	2 November 2009	Visual ID corroborated by S. Cerchio
AU-03	Exmouth	?	?	5.68	F	Juvenile	15 March 2015	Details to be provided in published note
MU-01	Diawling National Park	16º 32.4882' N	16º 27.0217' W	3.98	Unk	Juvenile	3 November 2013	Only Atlantic specimen known
PH-24	Pipol of Calayo, Nasugbu, Batangas	?	?	Unk	Unk	n/a	10 December 2009	Does not look like Omura's or Bryde's whale; 3 rostral ridges and both left and right lower jaw are white, S Cerchio
CI-01	Rarotonga	?	?	Unk	Unk	n/a	26 September 2000	Not confirmed, appearance distinctly different from Madagascar Omura's whales, S. Cerchio.

Literature Cited

1. Rosel, P. E. & Wilcox, L. A. 2014 Genetic evidence reveals a unique lineage of Bryde's whales in the northern Gulf of Mexico. *Endanger. Species Res.* **25**.

2. Yang, G., Liu, H., Zhou, K. & Ji, G. 2002 Identification of a *Balaenoptera edeni* specimen by using mitochondrial DNA sequences. *Chinese J. Zool.* **37**, 35–38.

3. Wada, S., Oishi, M. & Yamada, T. K. 2003 A newly discovered species of living baleen whale. *Nature* **426**, 278–281.

4. Sasaki, T., Nikaido, M., Wada, S., Yamada, T. K., Cao, Y., Hasegawa, M. & Okada, N. 2006 *Balaenoptera omurai* is a newly discovered baleen whale that represents an ancient evolutionary lineage. *Mol. Phylogenet. Evol.* **41**, 40–52. (doi:10.1016/j.ympev.2006.03.032)

5. Yoshida, H. & Kato, H. 1999 Phylogenetic relationships of Bryde's whale in the western North Pacific and adjacent waters inferred from mitochondrial DNA sequences. *Mar. Mammal Sci.* **15**, 1269–1286.

6. Yamada, T. K. et al. 2006 Middle-sized balaenopterid whale specimens (Cetacea: Balaenopteridae) preserved at several institutions in Taiwan, Thailand, and India. *Mem. Natl. Sci. Museum, Tokyo* **44**, 1–10.

7. Adulyanukosol, K., Thongsukdee, S., Passada, S., Prempree, T. & Wannarangsee, T. 2012 *Bryde's Whales in Thailand*. Bangkok: Aksornthai Printing Co.

8. Yamada, T. K., Kakuda, T. & Tajima, Y. 2008 Middle sized balaenopterid whale specimens in the Philippines and Indonesia. *Mem. Natl. Sci. Museum, Tokyo* **45**, 75–83.

9. Ponnampalam, L. S. 2012 Opportunistic observations on the distribution of cetaceans in the Malaysian South China, Sulu and Sulawesi Seas and an updated checklist of marine. *Raffles B Zool* **60**, 221–231.

10. Garrigue, C. & Poupon, M. 2013 *Guide d'identification: Mammifères Marins de Nouvelle-Calédonie*. Nouméa: Artypo.

11. Van Canneyt, O., Dorémus, G., Laran, S., Ridoux, V. & Watremez, P. 2015 REMMOA Nouvelle-Calédonie Wallis et Futuna: Rapport de campagne. *Rapp. intermédiaire pour l'Agence des Aires Mar. Protégées* , 65pp.

12. Yamada, T. K., Kemper, C., Tajima, Y., Umetani, A., Janetzki, H. & Pemberton, D. 2006 Marine Mammal Collections in Australia. *Natl. Sci. Museum Monogr.* **34**, 117–126.

13. Ottewell, K., Coughran, D., Gall, M., Irvine, L. & Byrne, M. In press. Stranding of Omura's Whale (*Balaenoptera omurai*) in Western Australia. *Aquat. Mamm.*

14. Jung, J. L., Mullié, W. C., Van Waerebeek, K., Wagne, M. M., Bilal, A. S. O., Sidaty, Z. A. O., Toomey, L., Méheust, E. & Marret, F. 2015 Omura's whale off West Africa: autochthonous population or inter-oceanic vagrant in the Atlantic Ocean? *Mar. Biol. Res.* **In Press**.
